# Atomically Precise Ag_11_ and Ag_12_ Nanocluster‐Assembled 2D Materials for Memristive and Neuromorphic Functionality

**DOI:** 10.1002/adma.74781

**Published:** 2026-08-26

**Authors:** Noohul Alam, Towhidur Rahaman, Céline Besnard, Arnulf Rosspeintner, Latevi Max Lawson Daku, Saood Khan, Soumya Jyoti Ray, Thomas Bürgi

**Affiliations:** ^1^ Department of Physical Chemistry University of Geneva Geneva Switzerland; ^2^ Department of Physics Indian Institute of Technology Patna Patna Bihar India; ^3^ Laboratory of Crystallography University of Geneva Geneva Switzerland

**Keywords:** artificial neural network, electronics, materials science, memristor, nanoclusters, nanotechnology, neuromorphic engineering, optoelectronics, resistive switching

## Abstract

Designing two‐dimensional (2D) materials from atomically precise nanocluster building blocks is a challenging task, yet crucial for next‐generation electronic devices. By tuning cluster nuclearity and ligand environment, well‐defined assemblies can be designed, enabling control over optoelectronic properties at the atomic level. Herein, we report the bottom‐up synthesis of two structurally distinct 2D nanocluster‐assembled materials constructed from Ag_12_ (2D Ag_12_‐CAM) and Ag_11_ (2D Ag_11_‐CAM) nanocluster building blocks. The change in cluster nuclearity arises solely from ligand tuning; benzenethiol yields the Ag_12_‐based framework, whereas 3,5‐bis(trifluoromethyl)benzenethiol (BTFMBT) directs formation of the Ag_11_ analogue. Lateral memristive devices fabricated from these materials (D‐1: 2D Ag_12_‐CAM; D‐2: 2D Ag_11_‐CAM) exhibit coexistence of negative differential resistance (NDR) and robust resistive switching with endurance stable over 4000 switching cycles. Device D‐2, based on 2D Ag_11_‐CAM, shows a markedly enhanced on‐off (*I*_on_/*I*_off_) ratio (∼ 642) compared to device D‐1 (∼ 200), and more pronounced NDR. Furthermore, the 2D Ag_11_‐CAM (D‐2)‐based device was evaluated for neuromorphic functionality, mimicking the adaptive behavior of biological synapses. It exhibits analogue potentiation and depression under pulse stimulation, while experimentally extracted synaptic weight updates enable an artificial neural network to achieve a test accuracy of 84.2%, approaching that of a machine learning (ML)‐trained network (93.2%).

## Graphical abstract

Two‐dimensional Ag_11_ and Ag_12_ cluster‐assembled materials (CAMs) are synthesized, offering atomically precise platforms with tunable electronic properties. The resulting materials exhibit robust memristive switching and neuromorphic response, demonstrating their promise for advanced nanoelectronic and memory applications.

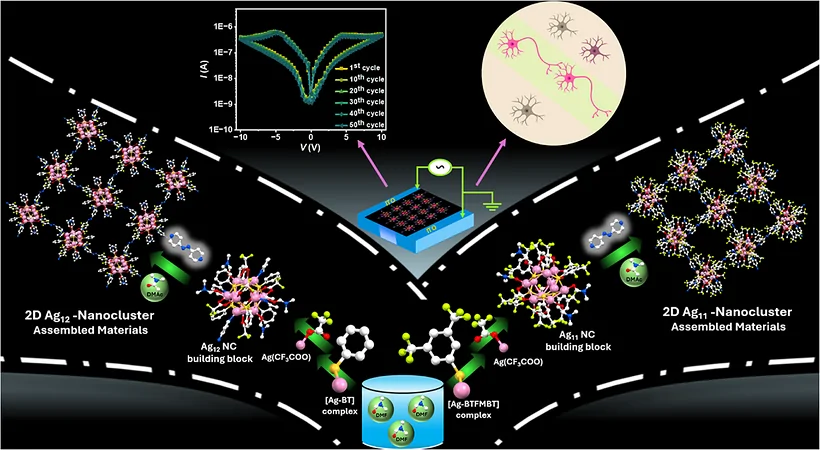

## 1 Introduction

Atomically precise, metal nanoclusters (NCs) bridge the gap between molecules and bulk metals [1, 2, 3, 4]. They feature discrete energy levels and well‐defined structures, enabling tunable properties through modifications to the core and surface ligands [4, 5, 6, 7]. However, despite their fascinating properties, the widespread use of isolated NCs remains limited, primarily due to their structural fragility and poor interparticle connectivity [8, 9, 10]. To overcome these limitations, a promising approach involves linking well‐defined nanocluster building blocks with organic linkers/metal centers to construct extended frameworks, thereby enhancing the stability of individual cluster nodes and imparting new collective properties that are inaccessible in the isolated state [11, 12, 13, 14, 15]. The resulting architecture can range from one‐ to three‐dimensional cluster‐assembled materials, depending on the coordination geometry and connectivity of the linkers [16, 17, 18, 19].

Two‐dimensional (2D) nanostructures, inspired by the breakthrough discovery of graphene and subsequent exploration of layered systems such as MoS_2_ have emerged as a particularly compelling class of materials [20]. Dimensional reduction profoundly alters the intrinsic properties of the materials [21]. However, many conventional 2D materials lack the modularity required for atomic‐level design, limiting precise control over structure–property relationships [22]. In contrast, 2D silver cluster‐assembled materials (2D Ag‐CAMs) offer unprecedented tunability through the rational choice of surface ligands and organic linkers, allowing systematic control over inter‐ and intra‐cluster interactions [23, 24, 25, 26]. By leveraging atomically defined building blocks, 2D Ag‐CAMs provide a chemically programmable platform for engineering low‐dimensional materials with tailored optoelectronic properties and tunable charge transport pathways, making them promising candidates for next‐generation electronic devices [24, 27, 28, 29, 30, 31]. Neuromorphic computing based on memristive synaptic devices has emerged as a promising route to energy‐efficient hardware implementation of synaptic plasticity, with analogue conductance modulation enabling direct weight updates in artificial neural networks [32, 33]. Materials that combine structural precision with tunable and nonvolatile resistive switching are therefore of particular relevance in this context [34]. Cluster‐based memristive switching, in which discrete metal clusters mediate resistance changes rather than extended conductive filaments, has demonstrated superior analogue linearity and conductance range in inorganic switching media [35]. However, the integration of 2D atomically precise cluster‐assembled frameworks as active memristive layers, where the cluster nuclearity, connectivity, and supramolecular organization are crystallographically characterized, represents a distinct and largely uncharted direction.

Given the unique advantages of atomically precise 2D Ag‐CAMs, we investigated their potential as active layers in negative differential resistance (NDR) controlled memristor devices [36]. Conventional memory technologies like dynamic random access memory (DRAM) require continuous power, and flash memories suffer from slower speeds and limited endurance. In contrast, NDR‐based memristor devices offer nonvolatile switching between high‐ and low‐resistance states under an electric field with additional control parameters [37, 38, 39, 40]. Insulating and wide‐bandgap materials are essential in such devices to enable stable and reproducible switching [41]. Resistive switching (RS) accompanied by specific physical phenomena, such as NDR, serves as a foundation for multifunctional devices [42, 43, 44]. However, lateral device configurations integrating both RS and NDR remain relatively underexplored. The coexistence of these two effects within a lateral architecture enables enhanced functional density and circuit‐level integration while preserving a compact footprint. Nonetheless, conventional memristive materials often suffer from uncontrolled defects and limited tunability, highlighting the need for atomically precise and structurally well‐defined systems [45, 46].

In this work, we have synthesized atomically precise two‐dimensional Ag‐based cluster‐assembled materials, 2D Ag_12_‐CAM and 2D Ag_11_‐CAM, constructed from well‐defined Ag_12_ and Ag_11_ nanocluster nodes (Figure 1). A small change in the thiolate ligand, benzenethiol versus 3,5‐bis(trifluoromethyl)benzenethiol, alters nuclearity and yields distinct 2D‐frameworks that allow direct comparison of their structural and electronic characteristics. The 2D Ag_11_‐CAM material shows stronger solid‐state emission and greater surface hydrophobicity than the Ag_12_ analogue. Lateral neuromorphic synaptic devices were fabricated using Ag_12_‐CAM (D‐1) and Ag_11_‐CAM (D‐2) as the active layer, exhibiting NDR‐assisted resistive switching. The devices maintain stable switching characteristics over 4000 consecutive cycles. Device D‐2 offers a wider conductance window with an *I*_on_/*I*_off_ ratio near 642, compared to about 200 for D‐1, which motivated its evaluation under pulsed operation. The 2D Ag_11_‐CAM based device exhibits clear potentiation and depression, demonstrating synaptic‐like modulation suitable for neuromorphic computing. Together, these results indicate that atomically precise silver cluster nodes offer a viable route to two‐dimensional materials that unite switchable transport and neuromorphic functionality.

**FIGURE 1:**
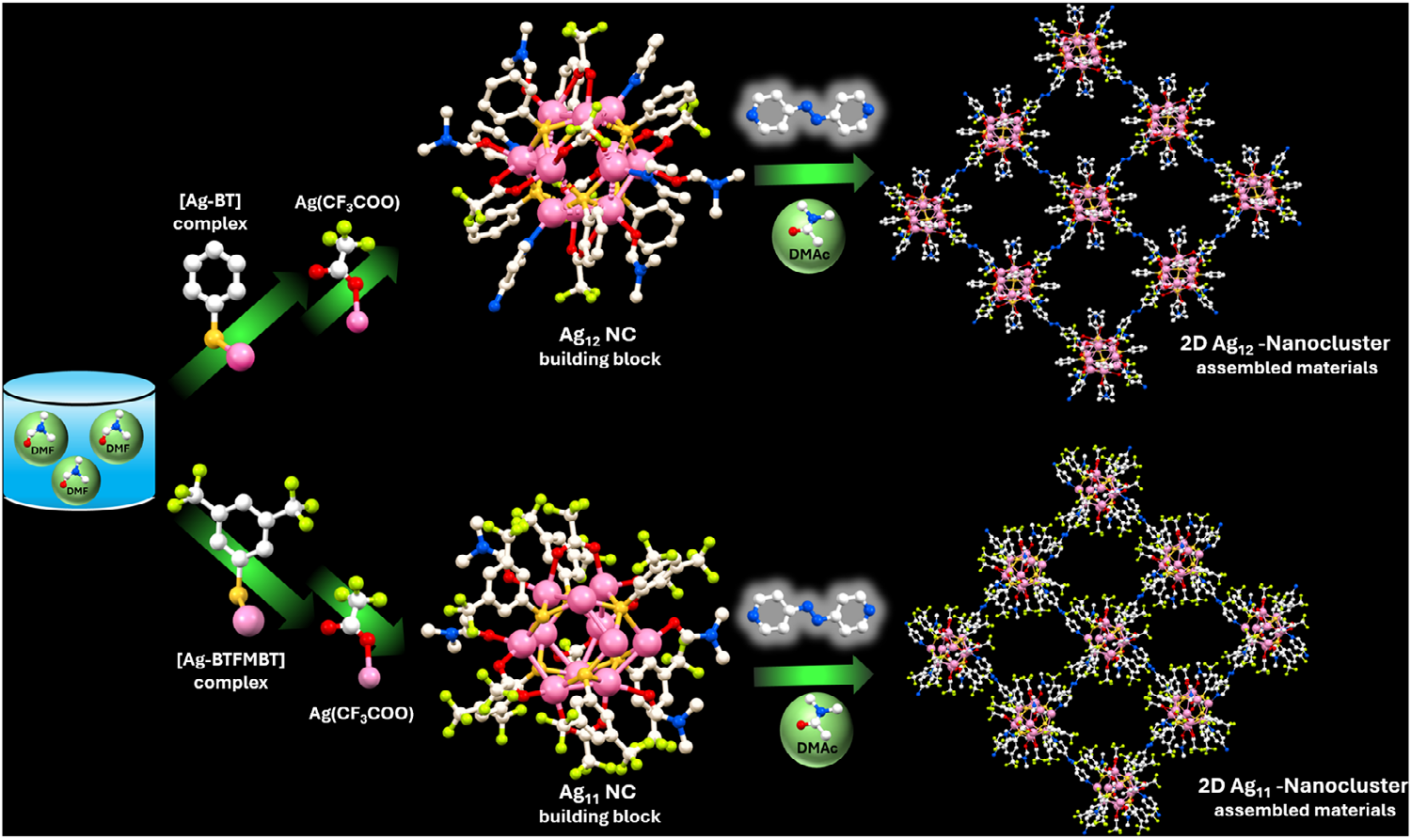
Schematic representation of the 2D silver cluster‐assembled materials formed from vertex‐deficient Ag_11_ and cuboctahedral Ag_12_ cores bridged by 4,4′‐azopyridine.

## 2 Results and Discussions

2D Ag‐CAMs were synthesized via a one‐pot, ambient‐condition chemical procedure. The 2D Ag_12_‐CAM was prepared by mixing a pre‐synthesized [Ag‐BT]n complex (0.1 mmol) and AgCF_3_COO (0.2 mmol) in a 1:2 molar ratio in 2 mL of DMF solution. Subsequently, 0.05 mmol of 4,4′‐Azopyridine (Azopy) linker in 1 mL of DMAc was carefully layered on top of the solution, leading to the formation of a 2D assembly bridged by the Azopy linker (Figure 1). This process resulted in red‐colored crystals of 2D Ag_12_‐CAM. The 2D Ag_11_‐CAM was synthesized by mixing a pre‐formed [Ag‐BTFMBT]n complex (0.05 mmol) (BTFMBT = 3,5‐bis(trifluoromethyl)benzenethiol) and AgCF_3_COO (0.1 mmol) in 2 mL of DMF, followed by careful layering of a DMAc solution (1 mL) containing the Azopy linker (0.05 mmol) (Figure 1). This process yielded red‐colored crystals of 2D Ag_11_‐CAM.

The single‐crystal x‐ray diffraction (SCXRD) analysis of 2D Ag_12_‐CAM crystals reveals that they crystallize in the monoclinic crystal system, adopting the *P*2_1_/c space group, with the repeating unit of [Ag_12_(C_6_H_5_S)_6_(CF_3_COO)_6_(C_3_H_7_NO)_4_(C_10_H_8_N_4_)_2_]_n_ (non‐coordinated lattice solvent omitted). The core of the cluster comprises twelve silver atoms arranged through argentophilic interactions with an average Ag···Ag distance of 3.22 Å (Figure 2a). This metallic kernel is stabilized by six benzenethiol ligands coordinated in a µ_4_‐η^1^:η^1^:η^1^:η^1^ mode with an average Ag─S bond distance of 2.48 Å (Figure 2b). The Ag_12_ core also incorporates four coordinated DMF solvent molecules (Figure S2a). In addition, six auxiliary trifluoroacetate (CF_3_COO^−^) ligands bridge the silver centers in a µ_2_‐η^1^:η^1^ fashion, with an average Ag‒O distance of 2.45 Å (Figure S3a). Each Ag_12_ node further connects to four Azopy linkers via Ag─N bonds (∼2.27 Å), forming a 2D extended periodic assembly by linking adjacent cluster nodes (Figures 2c,d and 3a). The inter‐cluster Ag···Ag distance between neighboring cluster nodes in the layer plane is measured to be 13.45 Å (Figure S4a). SCXRD analysis further reveals that the 2D Ag_12_‐CAM layers adopt an ABAB stacking sequence along the [001] direction, in which adjacent layers are laterally offset rather than directly eclipsed (Figure S5). Between adjacent layers, 2D Ag_12_‐CAM is stabilized primarily by C─F···H─C and C─O···H─C interactions (Figure S6).

**FIGURE 2:**
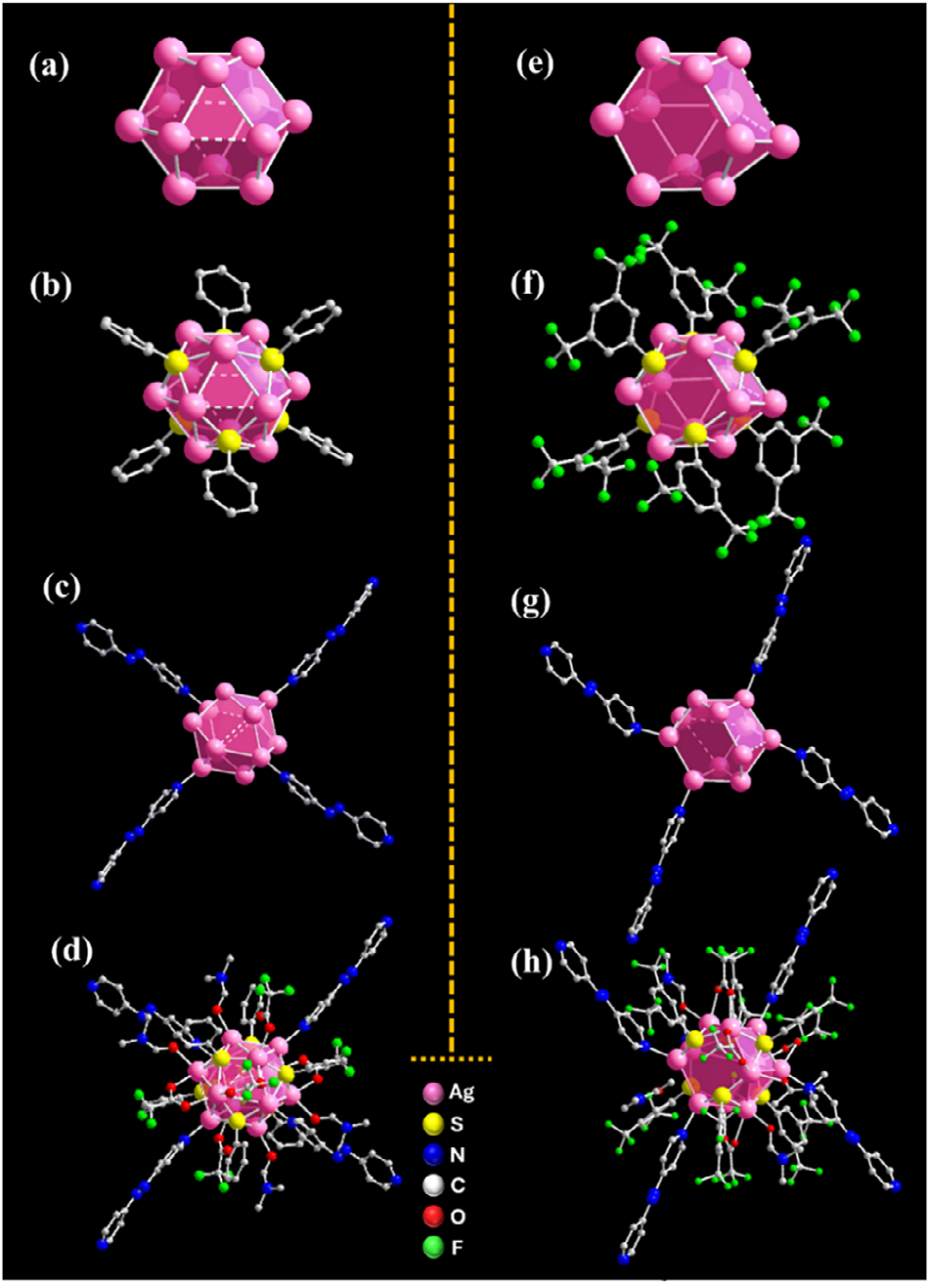
Structure of the cluster node of the 2D Ag_12_‐CAM and 2D Ag_11_‐CAM materials with protecting ligands and linkers. (a–d) 2D Ag_12_‐CAM: (a) Three planes forming the cuboctahedral Ag_12_ node, (b) six benzenethiol ligands on square facets, (c) Azopy linker coordination, (d) overall spatial arrangement of ligands and linkers. (e–h) 2D Ag_11_‐CAM: (e) Ag_11_ cluster node, highlighting the missing Ag atom from the hexagonal plane, (f) coordination of six 3,5‐bis(trifluoromethyl)thiophenol ligands, (g) four Azopy linkers bound to the Ag_11_ node, and (h) overall spatial arrangement of all ligands and linkers around the cluster node.

**FIGURE 3:**
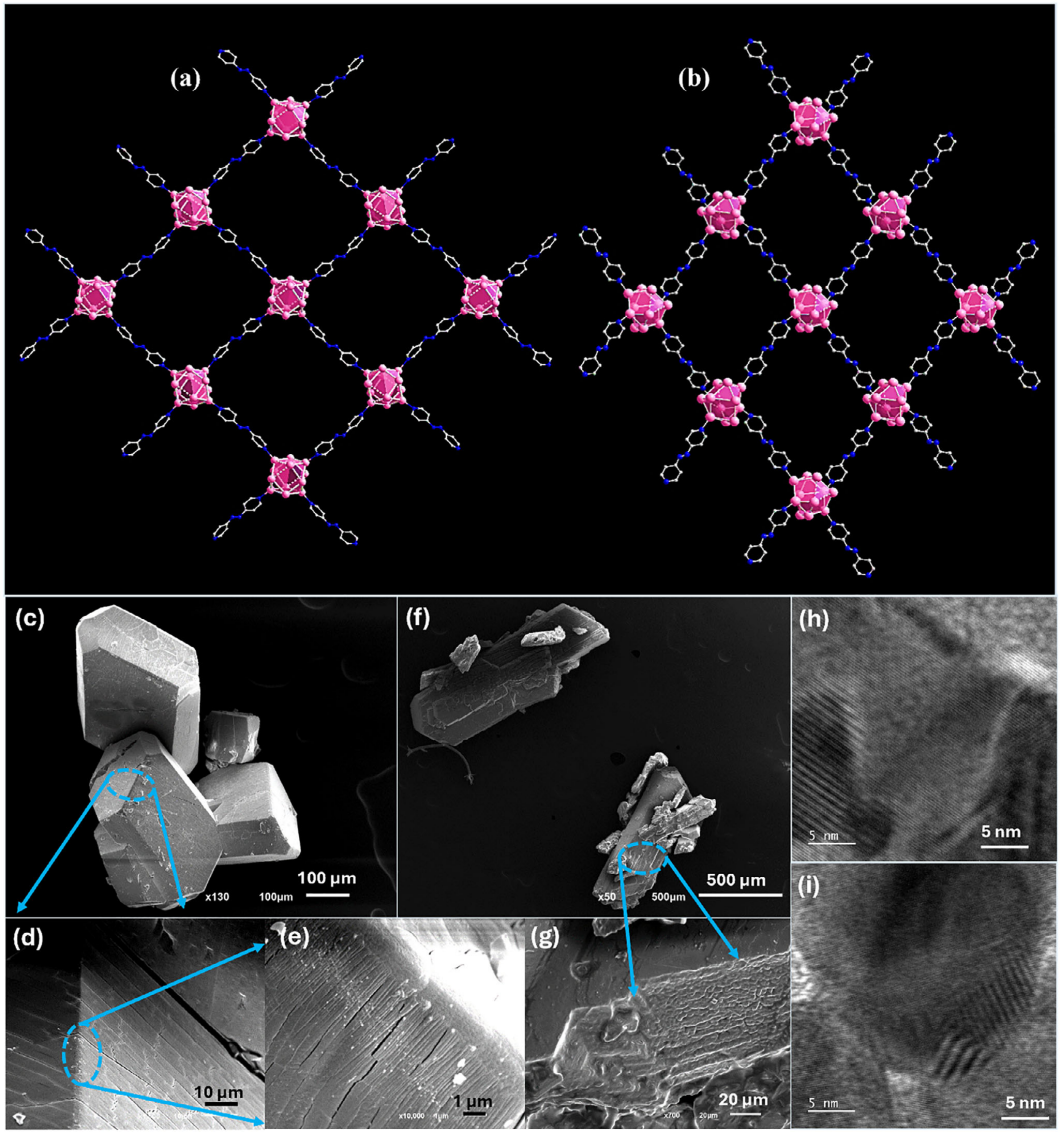
Planar 2D architecture with the cluster nodes connected by linker molecules: (a) 2D Ag_12_‐CAM, and (b) 2D Ag_11_‐CAM (for clarity, other ligands along with H atoms are omitted). (c) SEM micrograph of 2D Ag_12_‐CAM showing its block‐like morphology. (d,e) High‐magnification SEM images reveal that each block is composed of stacked 2D sheets. (f) SEM micrograph of 2D Ag_11_‐CAM, also displaying block‐like morphology. (g) High‐magnification SEM image confirming sheet‐like assembly within the blocks of 2D Ag_11_‐CAM. (h,i) HRTEM images of 2D Ag_12_‐CAM and 2D Ag_11_‐CAM, respectively, show well‐defined lattice fringes.

The 2D Ag_11_‐CAM, constructed from a fluorinated thiol ligand 3,5‐bis(trifluoromethyl)thiophenol, crystallizes in the monoclinic crystal system with the space group *P* 1 21 1 with the repeating unit of [Ag_11_(C_8_F_6_H_3_S)_6_(CF_3_COO)_5_(C_3_H_7_NO)_3_(C_4_H_9_NO)_1_ (C_10_H_8_N_4_)_2_]_n_. In contrast to its Ag_12_‐based analogue, this material features an Ag_11_ core, resulting from the absence of one silver atom in the middle atomic plane (Figure 2e, some residual electron density in the diffraction data may indicate minor Ag_12_ cores, with less than 14% abundance, Figure S1). The core now adopts a more open geometry, composed of five silver atoms in the equatorial layer and three atoms on both the top and bottom faces. This structural change is likely driven by the increased bulkiness and high fluorine content of the BTFMBT ligand, which may collectively destabilize the compact Ag_12_ arrangement. The modified nuclearity leads to a contraction in the average Ag···Ag distances (∼ 3.19 Å, removing disordered Ag positions for better accuracy), indicating that the incorporation of fluorine atoms enhances argentophilic interactions within the cluster. Notably, the Ag─S bond lengths increase to 2.51 Å compared to 2.48 Å in the non‐fluorinated thiol‐based 2D Ag_12_‐CAM (Figure 2f). Interestingly, among the six BTFMBT ligands coordinated to the Ag_11_ core, four adopt a µ_4_‐η^1^:η^1^:η^1^:η^1^ binding mode, while the remaining two exhibit a µ_3_‐η^1^:η^1^:η^1^ coordination. The core also accommodates three DMF and one DMAc solvent molecules, further stabilizing the open Ag_11_ architecture (Figure S2b). Despite the altered core, the structure maintains a 2D architecture, stabilized by five CF_3_COO^−^ ligands bridging in a µ_2_‐η^1^:η^1^ mode with Ag─O bond distance of 2.39 Å and four Azopy linkers coordinating via Ag─N bonds (∼2.45 Å), forming a well‐defined 2D extended network (Figure S3b, Figures 2g,h and 3b).

The average inter‐cluster Ag···Ag distance between neighboring cluster nodes in the planes is measured to be 13.38 Å (Figure S4b). The structure analysis shows that 2D Ag_11_‐CAM adopts an ABAB stacking sequence, with adjacent layers laterally offset relative to one another, as viewed along the [−1 0 −1] direction (Figure S7). 2D Ag_11_‐CAM exhibits a richer and more diverse supramolecular network, featuring C─F···F─C, C─F···H─C, and C─H···F─C interactions (Figure S8). Moreover, within each 2D sheet, 2D Ag_11_‐CAM also exhibits π–π stacking interactions between the Azopy linkers and BTFMBT ligands (average centroid–centroid distance: 3.67 Å) (Figure S9). This π–π stacking between the Azopy linker and the BTFMBT ligand, which is absent in 2D Ag_12_‐CAM, provides an additional mode of structural rigidification within the 2D sheet of 2D Ag_11_‐CAM, complementing the Ag─N coordination (∼2.45 Å) that links neighboring cluster nodes. The bulk phase purities of 2D Ag_12_‐CAM and 2D Ag_11_‐CAM were confirmed by powder X‐ray diffraction (PXRD) analysis, with experimental pattern matching with the simulated pattern derived from the SCXRD structure (Figure S10).

To gain insight into the electronic structure of the as‐synthesized 2D cluster‐assembled frameworks, DFT calculations were performed on finite cluster–linker–cluster models, in which two Ag_12_ (or Ag_11_) cluster nodes are bridged by a single Azopy linker, using coordinates taken directly from the SCXRD structures. In both models, the frontier occupied molecular orbitals (HOMO through HOMO‐4) are localized on the Ag_12_ (or Ag_11_) cluster cores, while the LUMO is centered on the Azopy linker (Figures S11 and S12), indicating that the unoccupied frontier states have linker character in both materials. The HOMO and LUMO energies are −5.134 and −4.233 eV for 2D Ag_12_–CAM, and −5.529 and −4.883 eV for 2D Ag_11_‐CAM, respectively.

Thermogravimetric analysis (TGA) was performed to assess the thermal stability of the as‐synthesized materials. 2D Ag_12_‐CAM exhibited sharp weight loss between 90°C and 165°C, attributed to the liberation of surface‐adsorbed moisture and solvent molecules within the crystal lattice (Figure S13). A sharper weight loss was observed between 230°C and 305°C, corresponding to the thermal decomposition of their frameworks. However, 2D Ag_11_‐CAM showed a more gradual mass loss in the lower temperature range, followed by a sharp weight loss between 230°C and 315°C. To probe the hydrophilicity of the materials, static water contact angle measurements were performed. The contact angle measurements revealed that 2D Ag_12_‐CAM exhibited contact angles of ∼70°, indicating greater surface wettability and hydrophilicity (Figure S14a). In contrast, 2D Ag_11_‐CAM demonstrated a contact angle of ∼139°, implying enhanced hydrophobicity and a lower affinity for water (Figure S14b). This increased hydrophobicity is likely due to the presence of the highly fluorinated BTFMBT ligand in 2D Ag_11_‐CAM, compared to the non‐fluorinated thiol ligands (i.e., benzenethiol) in 2D Ag_12_‐CAM.

Furthermore, scanning electron microscopy (SEM) was performed to examine the morphology of the as‐synthesized materials. At low magnification, the SEM micrographs reveal block‐like morphologies for both materials (Figure 3c,f). Higher‐magnification images reveal well‐defined layered architectures that stack atop one another, resulting in block‐like morphology of the synthesized 2D Ag_12_‐CAM and 2D Ag_11_‐CAM materials (Figure 3d,e,g). High‐resolution TEM images further confirm the crystalline nature of these layers, showing clear lattice fringes that validate the long‐range ordering within both 2D frameworks (Figure 3h,i).

The photophysical properties of both materials were characterized by diffuse reflectance spectroscopy (DRS) and photoluminescence measurements on single crystals. Kubelka–Munk analysis of the DRS data gives optical bandgaps of 2.16 eV for 2D Ag_12_‐CAM and 2.22 eV for 2D Ag_11_‐CAM (Figure 4a,b). Emission spectra were recorded from mounted single crystals under excitation at 2.58 eV (480 nm), using the same excitation power of 700 µW for the as‐synthesized materials (Figure 4c). 2D Ag_12_‐CAM shows a broad emission band spanning 1.2–2.0 eV, while 2D Ag_11_‐CAM displays a markedly more intense emission in the same spectral range (Figure 4d). DFT calculations on cluster–linker–cluster models show that the HOMO is localized on the Ag cluster core while the LUMO is centered on the Azopy linker in both systems (Figures S11 and S12), so the lowest‐energy transition has metal‐cluster‐to‐linker charge transfer (MLCT) character in both materials. Since both materials share this MLCT character and have similar optical bandgaps, the large difference in emission intensity is more likely to reflect a difference in the nonradiative decay rate between the two frameworks. We attribute this to the denser supramolecular network in 2D Ag_11_‐CAM, including π–π stacking between the Azopy linkers and BTFMBT ligands (3.67 Å, Figure S9) along with F···F, F···H, and H···H contacts (Figures S6 and S8), which may rigidify the cluster–linker geometry and limit nonradiative relaxation. This is similar to the cluster rigidification behavior reported for other luminescent metal nanocluster assemblies [47, 48, 49, 50]. The decay profile of 2D Ag_12_‐CAM is best represented by a power‐law function (Figure S15, see Supporting Information for more details). In contrast, the decay profile of 2D Ag_11_‐CAM is well described by a triple‐exponential model. The longest‐lived component (15.8 µs) represents the dominant contribution to the emission decay (Figure S16, see Supporting Information for more details).

**FIGURE 4:**
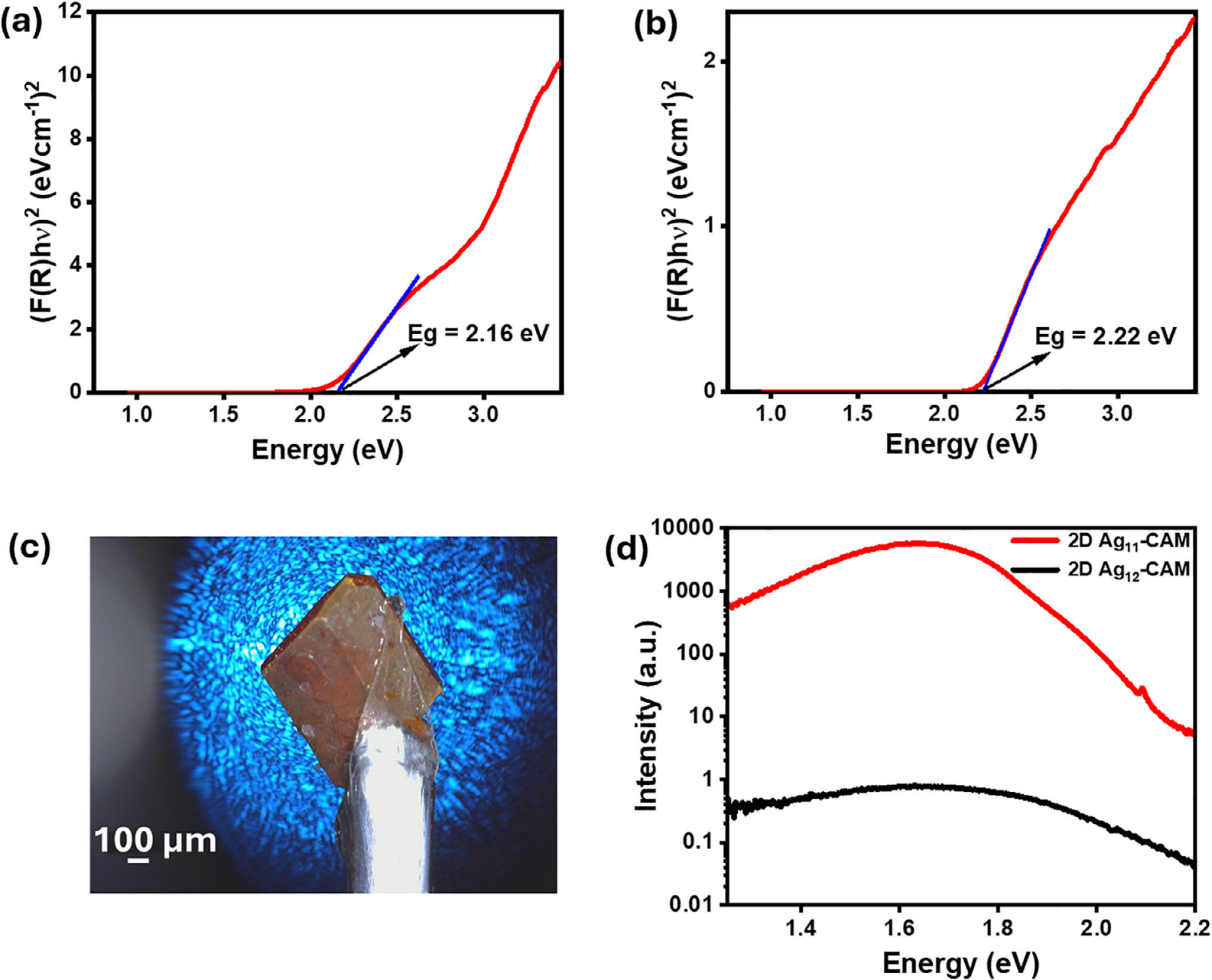
The optical bandgap of (a) 2D Ag_12_‐CAM and (b) 2D Ag_11_‐CAM, (c) Single crystal positioned on the measurement probe. (d) Emission spectra of 2D Ag_12_‐CAM and 2D Ag_11_‐CAM excited at 480 nm (2.58 eV) using an identical excitation power of 700 µW.

The atomically precise 2D architectures of as‐synthesized materials, along with subtle variations in their primary building units (i.e., Ag_12_ vs Ag_11_ cluster core), provide an ideal platform to investigate how discrete changes at the molecular level influence macroscopic electronic behavior. This level of precision and control is rarely attainable in conventional 2D inorganic materials, underscoring the promise of these cluster‐assembled 2D frameworks for next‐generation electronic applications. The electrical transport behavior of 2D Ag_12_‐CAM and 2D Ag_11_‐CAM was investigated by measuring the temperature‐dependent resistivity of pressed pellets using a two‐probe geometry and a cryogenic probe‐station setup. The electrical conductivities of 2D Ag_12_‐CAM and 2D Ag_11_‐CAM were calculated as 2.17 × 10^−6^ and 1.11 × 10^−5^ S/m, respectively, at 300 K. Both materials exhibit a negative temperature coefficient of resistivity (dρ/dT < 0) in the 250–300 K range, indicative of semiconducting behavior (Figure S17). Measurements below 250 K were not pursued due to increased noise and low current level. To examine the transport characteristics of 2D Ag_12_‐CAM and 2D Ag_11_‐CAM, the temperature dependence of the conductivity was analyzed using an Arrhenius model (Equation 1). Linear fits of ln σ versus 1000/*T* in the 250–300 K range yield activation energies of 0.78 and 0.60 eV for 2D Ag_12_‐CAM and 2D Ag_11_‐CAM, respectively (Figure 5a), confirming thermally activated transport in both materials

(1) $$\sigma \left(T\right)=\sigma_{0}\exp\left(-E_{a}/k_{B}T\right)$$

where the terms *σ*(T), σ_0_, *E*_a_, and *k*_B_ denote the conductivity at temperature *T*, the conductivity at *T* = 0 K, the carrier activation energy, and the Boltzmann constant, respectively.

**FIGURE 5:**
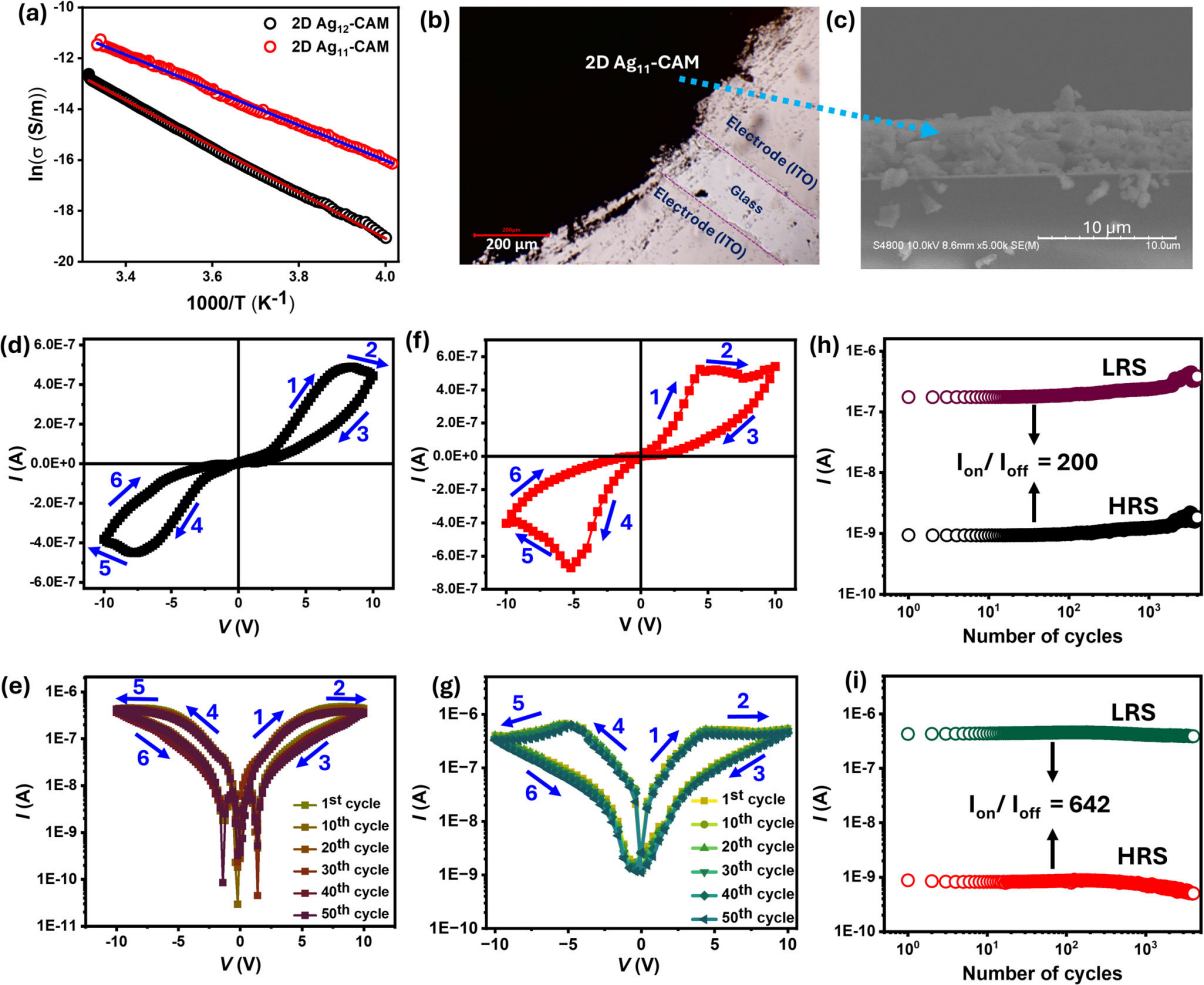
(a) Arrhenius plots of ln *σ* versus 1000/*T* (250–300 K) for 2D Ag_12_‐CAM and 2D Ag_11_‐CAM materials. (b) Optical microscopy image of a planar ITO‐based memristive device D‐2 under bottom illumination, where the active layer bridges an ITO channel separated by an exposed glass region. (c) The SEM image displays the cross‐sectional view of device D‐2, highlighting its layer structure. (d) *I*–*V* curve of device D‐1 (arrows indicate bias direction). (e) Hysteretic *I*–*V* curves of D‐1 for the first, 10th, 20th, 30th, 40th, and 50th cycles. (f) *I*–*V* curve of device D‐2 (arrows indicate bias direction). (g) Hysteretic *I*–*V* curves of D‐2 for the first, 10th, 20th, 30th, 40th, and 50th cycles. (h,i) Endurance tests of devices D‐1 and D‐2, respectively.

To gain deeper insight into the charge transport behavior, we have fabricated planar NDR memristive devices D‐1 and D‐2 based on 2D Ag_12_‐CAM and 2D Ag_11_‐CAM, respectively. The devices were fabricated by spin‐coating a slurry of the as‐synthesized 2D Ag_12_‐CAM and 2D Ag_11_‐CAM materials, made in isopropanol, onto pre‐patterned ITO electrodes on glass substrates (Figure 5b and Figure S18). PXRD patterns were recorded for the samples recovered after sonication in isopropanol for both materials (Figure S19). In both cases, the post‐sonication patterns retain the characteristic low‐angle reflections of the simulated structures, consistent with retention of the principal crystalline framework. Ag 3d XPS spectra recorded before and after device fabrication exhibit only minimal binding‐energy shifts (∼0.05 eV for 2D Ag_12_‐CAM and ∼0.18 eV for 2D Ag_11_‐CAM; Figure S20), consistent with preservation of the Ag chemical environment during device fabrication. A cross‐sectional SEM image of the slurry‐processed 2D Ag_11_‐CAM film (device D‐2) shows a compact and continuous layer formed by densely packed microcrystalline domains (Figure 5c). Subtle contrast variations and discernible boundaries between adjacent domains indicate the presence of interdomain interfaces generated during film formation through the packing of individual 2D Ag_11_‐CAM crystallites. Charge transport properties were evaluated using a two‐probe configuration under direct‐current (DC) bias. The applied voltage was swept sequentially in 0 V → 10V→ 0 V → −10 V → 0 V (as indicated by the arrows in the 1→6 path in Figure 5d) at a voltage sweep rate of 0.5 V s^−^^1^. The obtained I–V plot of device D‐1 displays nonlinear hysteretic behavior. Interestingly, the device exhibits resistive switching (RS) characteristics with two distinct features: diode‐like behavior in the low‐voltage region (−2 to +2 V) and NDR in the high‐voltage region (>4 V). During the forward voltage sweeps (Paths 1 and 3), the memristor device D‐1 switches from the low‐resistance state (LRS) to the high‐resistance state (HRS). Under the negative voltage sweeps (Paths 4 and 6), the device again transitions from LRS to HRS. To assess the stability of the hysteretic response, we performed measurements over 50 consecutive voltage‐sweep cycles, with every tenth cycle shown in Figure 5e. The retention of the hysteresis loop across all cycles confirms the robustness of the switching behavior. To evaluate the switching behavior in the low‐voltage region, the small‐signal regime was examined (Figure S21). During the forward sweep, the current remains higher under positive bias than under negative bias. In contrast, during the reverse sweep, the current increases under negative bias relative to the positive bias. This directional asymmetry in conduction establishes the diode‐like switching state of the device at low voltage. Further analysis of the charge‐transport behavior was carried out to understand the underlying conduction mechanism by using log |I| versus log |V| plots under both positive and negative bias (Figure S22). Changes in the slope across different voltage regions provide insight into the dominant conduction mechanisms operating in the device. A slope of ∼1 in the log |I|–log |V| plot indicates Ohmic conduction, while a slope near 2 corresponds to trap‐free space‐charge‐limited transport (SCLC) [37]. Slopes substantially greater than 2 are characteristic of the trap‐filled SCLC regime [37]. At the beginning of the forward sweep, Path 1 exhibits a slope of ∼1–2 (Figure 5d), indicating predominantly Ohmic conduction in the low‐bias region, with a gradual transition toward space‐charge‐limited conduction (SCLC) as shallow traps begin to fill (Figure S22). As the bias increases, Path 2 enters an NDR region, where carriers trapped in empty localized states create a space‐charge field, reducing the free carriers and suppressing current despite increasing voltage. Beyond this, Path 3 shows a slope of ∼2, consistent with trap‐free SCLC once the initial traps are saturated. During the reverse sweep, Path 4 retains a slope near 2, indicating continued SCLC behavior. Path 5 reveals a second NDR feature arising from voltage‐dependent trapping and de‐trapping of carriers. At higher negative bias in Path 6, the slope rises to ∼2.3, marking the trap‐filled SCLC regime in which deeper traps are progressively occupied, leading to a sharp increase in current once these states are saturated. This sequence of conduction events highlights how carrier trapping, trap filling, and intrinsic SCLC mechanisms orchestrate the complex current–voltage response of the device.

Similarly, the charge transport behavior of the D‐2 device was examined following a similar protocol as used for D‐1 to ensure a consistent comparison. The resulting *I*–*V* response of device D‐2 shows distinct HRS and LRS states (Figure 5f). Stability was verified over 50 consecutive sweeps (every 10th cycle in Figure 5g), which display consistent hysteresis behavior, confirming robust switching performance. Device D‐2 also shows RS behavior, with a diode‐like response at low voltage (–2 to +2 V) and an NDR response once the voltage exceeds ∼ 4 V. Notably, the NDR is significantly more pronounced than that of device D‐1. During the forward sweeps (Paths 1 and 3), the device switches from the LRS to the HRS. A similar LRS→HRS transition occurs during the negative‐bias sweeps (Paths 4 and 6). This symmetric response, showing both NDR and RS regardless of sweep direction, indicates unipolar switching behavior. To evaluate the switching behavior in the low‐voltage regime, we have isolated the small‐signal window (Figure S23). In this small region, the forward sweep gives a larger current at positive bias than at negative bias (Figure S23a). During the reverse sweep, the polarity dependence is inverted, and the current is larger at negative bias than at positive bias (Figure S23b). This polarity‐dependent response is consistent with diode‐like switching at low voltage. Logarithmic *I*–*V* plots of the D‐2 device were analyzed to elucidate the conduction mechanism (Figure S24). The linear fits reveal three distinct slopes, characteristic of space‐charge‐limited conduction (SCLC). In Path 1, at low bias, the conduction mechanism of the device follows Ohm's law (*I* ∝ *V*), which transitions to the Langmuir–Child law (*I* ∝ *V* ^1.5^) at higher voltage. Upon continuous sweeping under the same polarity, the slope (m) value exceeds 2, indicating a trap‐filled SCLC conduction mechanism (Path 3), as the injected carriers progressively occupy trap states within the 2D Ag_11_‐CAM film in the device. In the negative‐bias region, the device initially follows Ohmic conduction (Path 4). With continued sweeping (Path 6), the slope increases to 2.3, marking a transition to trap‐filled SCLC. Overall, these observations indicate that both materials exhibit thermally activated, trap‐mediated transport, but device D‐2 based on 2D Ag_11_‐CAM, demonstrates stronger trap involvement, reflected in both the steeper high‐bias slopes and the more prominent NDR features.

To quantify the NDR, we evaluated the peak‐to‐valley ratio (PVR = |*I*_peak_/*I*_valley_|) and the switching efficiency (SE = |(*I*_peak_ − *I*_valley_)/(*V*_peak_ − *V*_valley_)|), where the peak and valley correspond to the characteristic points in the *I*–*V* trace (Figure S25). Device D‐1 exhibits a maximum PVR of 1.20 and an SE of 34.5 nS, while device D‐2 shows an enhanced response with a PVR of 1.67 and an SE of 56 nS (Figure S25). These metrics indicate stronger conductance modulation and more efficient switching in device D‐2 based on 2D Ag_11_‐CAM. Mechanistically, NDR arises from voltage‐dependent trapping and de‐trapping of charge carriers at defect‐associated sites. At low bias, conduction is efficient, but beyond a threshold voltage, a substantial fraction of carriers becomes trapped, reducing the number of free carriers and suppressing current despite increasing bias. The stronger trap involvement in device D‐2, based on 2D Ag_11_‐CAM, aligns with the higher slopes in the SCLC regime and the more pronounced NDR, establishing it as a more effective platform for NDR‐assisted resistive switching. While such dynamic phenomena have been observed in quantum dots, 2D materials, and oxide memristors [51, 52, 53], their occurrence in 2D Ag‐cluster‐assembled materials is reported here for the first time. To evaluate endurance, both devices were subjected to 4000 consecutive switching cycles. No significant degradation was observed within the experimental range, with average *I*_on_/*I*_off_ ratios of 200 for D‐1 and 642 for D‐2 at a read voltage of 1 V (Figure 5h,i). These results indicate reliable and reproducible switching, demonstrating the suitability of as‐synthesized 2D Ag‐CAMs for memristor device fabrication. The measurable contrast between the ON and OFF states further highlights their potential for cost‐effective memory circuit design. To determine whether resistive switching originates from the assembled 2D framework rather than from its individual constituents, control devices were fabricated from the isolated [Ag‐BT]n complex, the isolated [Ag‐BTFMBT]n complex, and the Azopy linker alone, using the same device geometry and fabrication protocol as for D‐1 and D‐2. None of these components produced measurable *I*–*V* hysteresis when tested independently (Figure S26). Resistive switching was observed only for the fully assembled 2D Ag_12_‐CAM and 2D Ag_11_‐CAM frameworks, indicating that switching behavior requires the intact cluster–linker architecture rather than any single component in isolation. To assess fabrication reproducibility, electrical measurements were performed on 20 independently fabricated devices for each material. Of these, 15 devices based on 2D Ag_12_‐CAM and 17 devices based on 2D Ag_11_‐CAM exhibited hysteretic *I*–*V* characteristics (Figures S27 and S28).

The differences in device performance between D‐1 and D‐2 are consistent with the distinct transport properties and structural features of the corresponding frameworks. As discussed above, DFT calculations reveal a similar cluster‐centered HOMO and linker‐centered LUMO distribution for both 2D Ag_12_‐CAM and 2D Ag_11_‐CAM, indicating that the Azopy linker contributes to the low‐energy unoccupied electronic states in both materials. Despite this similar frontier‐orbital character, 2D Ag_11_‐CAM exhibits a lower activation energy for charge transport (0.60 eV) and a higher room‐temperature conductivity than 2D Ag_12_‐CAM. Consistent with these transport characteristics, device D‐2 displays a larger ON/OFF ratio, steeper high‐bias SCLC regions in the double‐logarithmic *I*–*V* plots, and more pronounced negative differential resistance than device D‐1.

Structurally, 2D Ag_11_‐CAM differs from 2D Ag_12_‐CAM through the presence of π–π stacking interactions between the Azopy linker and BTFMBT ligands (centroid–centroid distance = 3.67 Å), together with a richer network of F···F, F···H, and H···H contacts. Additional supramolecular interactions have been associated with enhanced charge‐transport and resistive‐switching characteristics in crystalline molecular materials [54, 55]. These structural features may therefore contribute to the more favorable transport characteristics and enhanced device performance observed for D‐2.

The retention characteristics of a memory device are critical for long‐term operational stability. To assess this, both D‐1 and D‐2 devices were monitored for up to 10^4^ s at room temperature (Figure S29). The *I*_on_/*I*_off_ ratios remained at 145 for D‐1 and 546 for D‐2 under a read voltage of 1 V, confirming the nonvolatile nature of the switching. To further evaluate thermal reliability, retention measurements were additionally performed at **333 K** under identical read conditions (Figure S29). While D‐1 exhibits a gradual decrease in the ON‐state current after prolonged operation, D‐2 maintains a well‐defined resistance window throughout the measurement despite minor fluctuations in the OFF‐state current. These results demonstrate the superior thermal retention performance of the 2D Ag_11_‐CAM‐based device. In addition, D‐1 and D‐2 were remeasured after prolonged ambient exposure (>5 months). Both devices preserved their characteristic NDR‐assisted resistive‐switching behavior, further demonstrating the long‐term stability of the memristive devices under ambient laboratory conditions (Figure S30).

In NDR‐based memristive systems, the peak‐to‐valley ratio (PVR) defines the extent of current contrast and strongly influences oscillatory dynamics in neuromorphic architectures. The increase in PVR from 1.20 in D‐1 to 1.67 in D‐2 improves ON/OFF discrimination, thereby enhancing noise tolerance and multilevel stability, consistent with recent NDR‐based neuromorphic systems [56, 57, 58]. In parallel, the higher switching efficiency of D‐2 (56 vs. 34.5 nS) reflects a steeper negative differential conductance, facilitating faster switching dynamics and spike generation [56]. Together, these improvements identify D‐2 as the more capable device, motivating its selection for synaptic evaluation. In biological synapses, signal transmission strength evolves continuously based on prior stimuli, and such behavior is most effectively mimicked in electronic systems when the conduction mechanism is governed by progressive charge trapping and detrapping [59, 60]. To probe this behavior experimentally, we applied sequential excitatory voltage pulses (1 V, 0.20 s), which elicited a steady increase in postsynaptic current, indicative of synaptic potentiation (Figure S31). Subsequent application of inhibitory pulses induced a monotonic decrease in conductance, representing synaptic depression (Figure S31). This bidirectional, analogue tuning of conductance closely mimics the adaptive plasticity of biological synapses and confirms the suitability of 2D Ag_11_‐CAM for neuromorphic operation.

To evaluate learning performance, the experimentally measured potentiation–depression profiles were incorporated into an artificial neural network (ANN) for an off‐chip image‐recognition task using the Modified National Institute of Standards and Technology (MNIST) dataset (Figure 6a). A single‐layer ANN was employed and trained over 100 epochs (see Supporting Information for hyperparameters and implementation details, Table S3). The device‐enabled ANN achieves a test accuracy of 84.2%, comparable to the purely software‐trained model (93.2%), demonstrating that the analogue, nonvolatile weight modulation of D‐2 effectively supports hardware‐level learning (Figure 6b). These results underscore the potential of 2D Ag_11_‐CAM‐based memristors as compact and energy‐efficient building blocks for future neuromorphic computing architectures. To further evaluate the robustness of the proposed hardware implementation, the ANN model was extended by incorporating additional experimentally measured device characteristics, including cycle‐to‐cycle variability and asymmetric potentiation/depression characteristics (Figure S33). In addition, sparse‐coding simulations demonstrate accurate image reconstruction, further highlighting the versatility of the proposed memristive synapse for neuromorphic computing (Figure S34). These results underscore the potential of 2D Ag_11_‐CAM‐based memristors as compact building blocks for future neuromorphic computing architectures.

**FIGURE 6:**
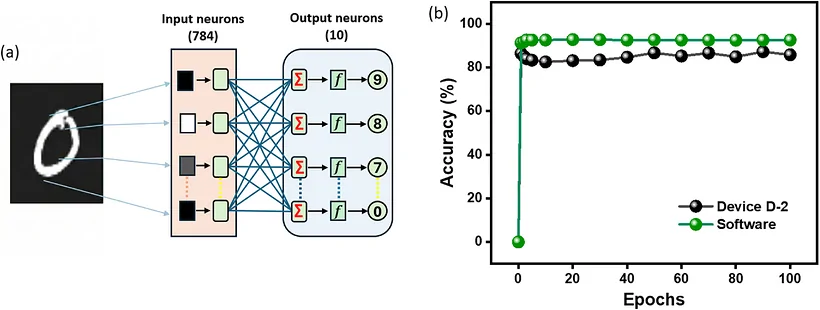
(a) Schematic of the neural network used for MNIST pattern recognition and corresponding weight distribution, and (b) accuracy evaluation of the quantization methods as a function of the number of training epochs.

## 3 Conclusion

In summary, we show that a subtle modification in the thiolate ligand directs the formation of two distinct Ag_12_‐ and Ag_11_‐based building units, giving rise to atomically precise 2D cluster‐assembled materials. The resulting 2D Ag_12_‐CAM and 2D Ag_11_‐CAM frameworks provide a chemically well‐defined platform to probe how cluster nuclearity and surface chemistry influence solid‐state optical response and charge transport. Distinct differences in emission behavior and surface wettability arise directly from the underlying cluster structures. Lateral memristive devices fabricated from these materials reveal that both systems support NDR and stable resistive switching, with the 2D Ag_11_‐CAM device (D‐2) achieving a substantially higher *I*_on_/*I*_off_ ratio (∼ 642) than its Ag_12_ analogue (∼ 200). The enhanced transport characteristics of D‐2 further enable reliable synaptic behavior under pulsed excitation, including analogue potentiation and depression, and support hardware‐level learning with high classification accuracy. Moreover, the device maintains stable resistive‐switching behavior after prolonged ambient storage (>5 months) and retains a well‐defined resistance window during retention measurements at 333 K, demonstrating its operational robustness under practical conditions. Hardware‐aware simulations incorporating additional experimentally measured device characteristics further confirm the robustness of the proposed memristive synapse for neuromorphic computing. These findings establish 2D Ag cluster‐assembled materials as a promising platform for integrating atomically precise structure with functional nanoelectronic behavior, offering new opportunities for designing cluster‐based materials for future memory and neuromorphic technologies.

## Author Contributions

**Noohul Alam**: conceptualization, investigation, writing – original draft, data curation. **Towhidur Rahaman**: conceptualization, investigation, data curation, writing – original draft. **Céline Besnard**: investigation, writing – review and editing, data curation. **Arnulf Rosspeintner**: investigation, writing – review and editing, data curation. **Latevi Max Lawson Daku**: investigation, validation, data curation. **Saood Khan**: investigation, writing – review and editing. **Soumya Jyoti Ray**: conceptualization, writing – review and editing, supervision, methodology. **Thomas Bürgi**: conceptualization, funding acquisition, writing – review and editing, supervision.

## Conflicts of Interest

The authors declare no conflicts of interest.

## Supporting Information

CCDC 2531411 and 2531412 contain the supplementary crystallographic data for 2D Ag_12_‐CAM and 2D Ag_11_‐CAM, respectively. These data can be obtained free of charge from The Cambridge Crystallographic Data Centre via www.ccdc.cam.ac.uk/structures.

## Supporting information




**Supporting File**: adma74781‐sup‐0001‐SuppMat.docx.

## Data Availability

All data generated or examined during this study are provided within this article and its Supporting Information. Any additional supporting data can be obtained from the corresponding authors upon reasonable request.
