# Supplementary material for: Atomically Precise Ag11 and Ag12 Nanocluster‐Assembled 2D Materials for Memristive and Neuromorphic Functionality
Source: Adv Mater. 2026 Aug 26;38(59):e74781. doi: 10.1002/adma.74781 (PMC13689899; doi:10.1002/adma.74781)
Supplement: Supplementary file 1 [file ADMA-38-e74781-s001.docx]

**Supporting Information for**

**Atomically Precise Ag_11_ and Ag_12_ Nanocluster-Assembled 2D Materials for Memristive and Neuromorphic Functionality**

Noohul Alam,^#^*^[1]^ Towhidur Rahaman,^#[2]^ Céline Besnard,^[3]^ Arnulf Rosspeintner, ^[1]^ Latevi Max Lawson Daku,^[1]^ Saood Khan,^[2]^ Soumya Jyoti Ray,*^[2]^ and Thomas Bürgi*^[1]^

[1]. Department of Physical Chemistry, University of Geneva, 30 Quai Ernest-Ansermet, 1211 Geneva, Switzerland.

[2]. Department of Physics Indian Institute of Technology Patna, Bihar, 801106, India.

[3]. Laboratory of Crystallography, University of Geneva, Quai Ernest Ansermet 24, 1211 Geneva, Switzerland.

[#] Equal contribution by these authors.

***Correspondence to**: [Thomas.Buergi@unige.ch](mailto:Thomas.Buergi@unige.ch), [Ray@iitp.ac.in](mailto:Ray@iitp.ac.in) and [Noohul.Alam@unige.ch](mailto:Noohul.Alam@unige.ch)

**Table of contents**

| **General methods** | S5 |
| --- | --- |
| **Table S1**. Crystal data and structure refinement for 2D Ag_12_-CAM. | S10 |
| **Table S2**. Crystal data and structure refinement for 2D Ag_11_-CAM. | S12 |
| ***Figure S1.*** *(a) OMIT map showing the residual density on the potential low-occupancy extra Ag atom. (b) OMIT map showing the electronic density on the DMAc molecule close to the possible extra Ag position.* | S14 |
| **Figure S2.** (a) Four DMF molecules are coordinated to the Ag_12_ core of 2D Ag_12_-CAM, and (b) three DMF molecules and one DMAc molecule are coordinated to the Ag_11_ core of 2D Ag_11_-CAM. | S16 |
| **Figure S3**. The auxiliary ligand CF_3_COO− connected with (a) Ag_12_ core of 2D Ag_12_-CAM, and (b) Ag_11_ core of 2D Ag_11_-CAM. | S16 |
| **Figure S4**. The Azopy linker is connected with (a) Ag_12_ core of 2D Ag_12_-CAM, and (b) Ag_11_ core of 2D Ag_11_-CAM. | S17 |
| **Figure S5.** Interlayer stacking mode of 2D Ag_12_-CAM. (a) View showing the stacking of adjacent layers in an ABAB sequence. (b) View along [001] showing the lateral displacement of layer B (green) relative to layer A (blue). | S17 |
| **Figure S6.** Adjacent layers of the 2D Ag_12_-CAM are interconnected mostly through C–F···H–C (~2.60 Å) and C–O···H–C (~2.68 Å) interactions, as analyzed using Mercury software. | S18 |
| **Figure S7.** Interlayer stacking mode of 2D Ag_11_-CAM. (a) View showing the stacking of adjacent layers in an ABAB sequence. (b) View along [-1 0 -1] showing the lateral displacement of layer B (green) relative to layer A (blue). | S18 |
| **Figure S8**. Adjacent layers of the 2D Ag_11_-CAM are interconnected mostly through C–F···H–C (~2.41 Å), C–F···F–C (~2.82 Å), and C–H···H–C (~2.17 Å) interactions, as analyzed using Mercury software. | S19 |
| **Figure S9**. Structural view of π–π stacking interactions within each sheet of 2D Ag_11_-CAM between the aromatic ring of Azopy linkers and BTFMBT ligands, showing an average centroid–centroid distance of 3.67 Å, which contributes to framework stabilization (analyzed by using Olex2 software). | S21 |
| **Figure S10**. Powder X-ray diffraction pattern of (a) 2D Ag_12_-CAM, *(b)* *2D Ag_11_-CAM*. | S20 |
| ***Figure S11****. Molecular orbital energy diagram of the cluster–linker–cluster model representing 2D Ag_12_-CAM, showing the spatial distribution of selected frontier orbitals (HOMO to HOMO-4 and LUMO to LUMO+2).* | S21 |
| ***Figure S12****. Molecular orbital energy diagram of the cluster–linker–cluster model representing 2D Ag_11_-CAM, showing the spatial distribution of selected frontier orbitals (HOMO to HOMO-4 and LUMO to LUMO+2).* | S22 |
| **Figure S13**. TGA analysis of 2D Ag_12_-CAM and 2D Ag_11_-CAM. | S23 |
| **Figure S14**. Contact angle measurement for (a) 2D Ag_12_-CAM and (b) 2D Ag_11_-CAM. | S23 |
| ***Figure S15****: Luminescence decay of 2D Ag_12_-CAM and the corresponding power-law fit. The upper panel shows the weighted residuals.* | S24 |
| ***Figure S16****: Luminescence decay of 2D Ag_11_-CAM and the corresponding triple-exponential fit. The upper panel shows the weighted residuals*. | S24 |
| **Figure S17**. Variation of resistivity as a function of temperature for (a) 2D Ag_12_-CAM and (b) 2D Ag_11_-CAM. | S25 |
| **Figure S18**. Patterned ITO/glass substrate showing two parallel ITO electrodes separated by an etched glass gap (~200 μm), defining the lateral device geometry. | S25 |
| ***Figure S19****. Comparison of the simulated PXRD patterns of (a) 2D Ag_12_-CAM and (b) 2D Ag_11_-CAM with those of the corresponding samples recovered after 1 h sonication in isopropanol. Intensities were normalised to the most intense diffraction peak for ease of comparison.* | S26 |
| ***Figure S20****. High-resolution Ag 3d XPS spectra of (a) 2D Ag_12_-CAM and (b) 2D Ag_11_-CAM recorded before and after device fabrication. High-resolution O 1s XPS spectra of (a) 2D Ag_12_-CAM and (b) 2D Ag_11_-CAM recorded before and after device fabrication.* | S27 |
| **Figure S21**. Small-signal I–V characteristics of device D-1: (a) Forward-sweep small signal I–V curves exhibiting pronounced current asymmetry favouring positive bias. (b) Reverse-sweep small-signal I–V curves displaying stronger conduction under negative bias. The bias-dependent asymmetry captured in both figures unambiguously demonstrates diode behaviour. | S28 |
| **Figure S22**. Double-logarithmic plots with corresponding linear fits for device D-1: (a) Path 1 during the forward voltage sweep, (b) Path 3 in the forward sweep, (d) Path 4 in the reverse sweep, (e) Path 6 in the reverse sweep. (c) Negative differential resistance (NDR) behavior under positive bias, and (f) NDR behavior under negative bias. | S28 |
| **Figure S23**. Small-signal I–V characteristics of device D-2: (a) Forward-sweep small signal I–V curves exhibiting pronounced current asymmetry favouring positive bias. (b) Reverse-sweep small-signal I–V curves displaying stronger conduction under negative bias. The bias-dependent asymmetry captured in both figures unambiguously demonstrates diode behaviour. | S29 |
| **Figure S24**. Double-logarithmic plots with corresponding linear fits for device D-2 (a) Path 1 during the forward voltage sweep, (b) Path 3 in the forward sweep, (d) Path 4 in the reverse sweep, and (e) Path 6 in the reverse sweep. (c) NDR behavior under positive bias, and (f) NDR behavior under negative bias. | S29 |
| **Figure S25**. I–V plot illustrating peak-to-valley ratio (PVR) and switching efficiency (SE) of the devices (a) D-1 and (b) D-2. | S30 |
| ***Figure S26.*** *Current–voltage (I–V) characteristics of control devices fabricated from the individual framework components: (a) Ag–BT complex, (b) Ag–BTFMBT complex, and (c) Azopy linker, measured under the same device geometry and fabrication conditions as those used for devices D-1 and D-2. Measurements were reproduced using multiple independently fabricated control devices.* | S30 |
| ***Figure S27.*** *Device-to-device reproducibility of the 2D Ag_12_-CAM (D-1) memristive devices. Representative current–voltage (I–V) characteristics of 20 independently fabricated devices measured under identical fabrication and testing conditions* | S31 |
| ***Figure S28****. Device-to-device reproducibility of the 2D Ag_11_-CAM (D-1) memristive devices. Representative current–voltage (I–V) characteristics of 20 independently fabricated devices measured under identical fabrication and testing conditions.* | S31 |
| ***Figure S29.*** *Retention characteristics of (a) D-1 and (b) D-2 measured at room temperature (RT) and 333 K as a function of time using a 1 V read pulse with a pulse width of 200 ms.* | S32 |
| ***Figure S30.*** *Long-term ambient stability of the memristive devices after storage under ambient laboratory conditions (22–27 °C) for more than five months. (a,b) Resistive-switching characteristics and (c,d) corresponding semi-logarithmic current–voltage (I–V) curves of devices D-1 and D-2 measured after prolonged ambient exposure.* | S32 |
| **Figure S31**. Current behaviour of the 2D Ag_11_-CAM with respect to the number of identical pulses and its fit curve using a model Eq. S5. | S35 |
| **Figure S32**. Accuracy evaluation of the quantization methods with the variation of the input MNIST image resolutions. | S36 |
| ***Figure S33****. Neuromorphic performance of the 2D Ag_11_-CAM-based device incorporating experimentally measured device non-idealities. (a) Experimentally measured potentiation/depression characteristics used for hardware-aware ANN simulations. (b) Cycle-to-cycle conductance variability extracted from repeated synaptic updates. (c) Comparison of software accuracy and hardware-aware accuracy after incorporating experimentally measured device non-idealities. (d) Confusion matrix for MNIST classification obtained using the experimentally informed hardware mode* | S37 |
| ***Figure S34.*** *Comparison of original MNIST digit images with their corresponding software-based and memristor-mapped sparse coding reconstructions. The dictionary was trained offline using sparse coding on non-overlapping 4 × 4 image patches extracted from the MNIST training dataset and subsequently mapped onto experimentally measured memristor conductance states using the experimentally derived LTP/LTD characteristics. Six representative examples of the digit '3' are shown. The mean squared error (MSE) between each reconstructed image and its corresponding original image is indicated above the reconstructed images. The memristor-mapped implementation exhibits a slightly higher MSE than the software implementation owing to conductance quantization during hardware mapping.* | S38 |
| Artificial Neural Network training and MNIST recognition. | S33 |
| Conductance Model and Weight Mapping | S33 |
| Accuracy and Resolution Dependence | S34 |
| *Hardware-Aware ANN Evaluation.* | S36 |
| *Sparse-coding image reconstruction.* | S36 |
| *Energy Consumption.* | S38 |
| **Table S3**. Hyperparameters and their description used for ANN simulations | S39 |
| **Table S4**. Benchmark comparison of the neuromorphic performance of the as-synthesised 2D Ag_11_-CAM memristive devices with representative Ag-based cluster materials, metal–organic framework (MOF)-based, oxide-based, and other low-dimensional memristive systems reported in the literature. | S40 |
| **References** | S41 |

**General methods:**

**Materials.** Benzenethiol (BT), 3,5-Bis(trifluoromethyl)benzenethiol (BTFMBT), silver trifluoroacetate (CF_3_COOAg), silver nitrate (AgNO_3_), 4,4'-azopyridine (Azopy), and potassium bromide (KBr) were purchased from Sigma-Aldrich. HPLC-grade solvents: methanol, dimethylformamide (DMF), and dimethylacetamide (DMAc) were also purchased from Sigma-Aldrich. Triethylamine was bought from Fluorochem.

**Synthesis of silver-thiolate complexes and 2D Ag-CAMs.**

**Synthesis of [Ag-BT]_n_ complex.** Silver nitrate (10 mmol) and benzene thiol (30 mmol, 1:3 molar ratio) were mixed in 40 mL of methanol solvent. Triethylamine (400 µL) was added dropwise under stirring, and the mixture was stirred at room temperature for 24 h. A pale-yellow precipitate formed, which was collected by filtration, washed with cold methanol, and dried under vacuum to give the [Ag-BT]_n_ complex.

**Synthesis of [Ag-BTFMBT]_n_ complex.** [Ag-BTFMBT]_n_ complex was prepared using the same procedure as above, replacing benzene thiol with 3,5-bis(trifluoromethyl)benzenethiol (BTFMBT), using an AgNO_3_:BTFMBT molar ratio of 1:3. Triethylamine (500 µL) was added, and the mixture was stirred at room temperature for 24 h. The resulting pale-yellow precipitate was collected, washed with cold methanol, and dried under vacuum.

**Synthesis of 2D Ag_12_-CAM.** The [Ag-BT]_n_ complex (0.1 mmol) and AgCF_3_COO (0.2 mmol) were dissolved in 2 mL of DMF. Separately, 0.05 mmol of 4,4′-Azopyridine (Azopy) was dissolved in 1 mL of DMAc and carefully layered on top of the DMF solution. The vial was kept at room temperature, and red-colored crystals of 2D Ag_12_-CAM formed within one week.

**Synthesis of 2D Ag_11_-CAM.** The [Ag-BTFMBT]_n_ complex (0.05 mmol) and AgCF_3_COO (0.1 mmol) were dissolved in 2 mL of DMF. Separately, 0.05 mmol of Azopy in 1 mL of DMAc was layered on top of the DMF solution. Red-colored crystals of 2D Ag_11_-CAM formed within one week at room temperature under ambient conditions.

**Instrumentation.** The scanning electron microscope (SEM - Jeol 6510LV) was used for the microscopic characterization of the as-synthesized 2D Ag_12_-CAM and 2D Ag_11_-CAM materials. SEM images of device D-2 (ITO/2D Ag_11_-CAM/ITO) were acquired using a **Zeiss GeminiSEM 300 field-emission scanning electron microscope.** Thermal characterization was carried out using a NETZSCH STA 449 F3. Alumina crucibles were used for all experiments. The analyses were carried out under a nitrogen atmosphere with a constant flow rate of 80 mL/min with a heating rate of 10 K/min. Powder X-ray measurements were performed using a Panalytical X’Pert diffractometer with Cu Kα1 radiation (1.540 Å). A Krüss EasyDrop goniometer (DSA-20E) was used to measure the contact angles of the materials*.* Sonication was performed to prepare the slurries using an HV-PRO-650 probe sonicator (Hoverlabs) equipped with a 10 mm probe. The slurries were sonicated at 30% amplitude for 1 h in pulse mode (2 s on/2 s off) under ice-bath cooling. TEM micrographs were recorded using a JEOL 2100F FETEM instrument. XPS measurements were performed using a Physical Electronics Versa Probe III system. All XPS spectra were calibrated using the adventitious carbon C 1s peak at 284.8 eV as the binding-energy reference.

X-ray crystallography details. Suitable crystals of 2D Ag_12_-CAM and 2D Ag_11_-CAM were selected and X-ray intensity data were collected on a Rigaku XtaLAB Synergy, Dualflex, HyPix-Arc 150° diffractometer using Cu Kα radiation (λ = 1.54184 Å). Using Olex2,^[1]^ the structures were solved with the SHELXT^[2]^ structure solution program using dual space methods and refined with the SHELXL^[2]^ refinement package using Least Squares minimization. Summaries of crystal data and structure refinement are given in Tables 1 and Table 2.

**Photophysical Studies.** Luminescence measurements (time-resolved and steady-state) were performed on a home-built set-up.

Excitation. The light sources were two broadband light-sources (NKT SuperK Extreme and SuperK Compact) in combination with an acousto-optic transmission filter (NKT SuperK Select). The polarization of the excitation was controlled using a Glan-Taylor polarizer and was set to s-polarization.

Light collection. Fluorescence of the samples was collected using a Cassegrain type collection optic (Anagrain; Anaspec Research Laboratories Ltd., Berkshire, UK) in 180-back-scattering geometry, passed through appropriate long-pass filters (if necessary), a wire-grid polarizer (Moxtek UBB01A) at magic angle with respect to the excitation, and focused onto a fiber/fiber-bundle (round-to-linear) using an off-axis parabolic mirror (Thorlabs, MPD149-P01).

Vis-detection. A home-built double-prism spectrograph (courtesy by Niko Ernsting) in combination with a Pixis 2K camera (Princeton Instrument) was used to disperse and detect the luminescence in the range 350-1050nm.^[3]^ All spectra were corrected for the detection sensitivity. NIR-detection. An Andor Shamrock 163 spectrograph in combination with an Andor iDUS 490 InGaAs 1.7 camera was used to disperse and detect the luminescence in the range 750-1600nm (100lines/mm grating, 780nm blaze). A custom made variable longpass filter (Materion) was used to suppress second-order artifacts. All spectra were corrected for the detection sensitivity.

Time-Resolved detection: The fiber output was focused on a single-photon counting APD (MPD-100-CTB) using an ellipsoidal mirror (Horiba, 1427C). A variety of longpass and bandpass filters were used for the wavelength selection. The time-correlated photon counting was performed with a Multiharp-150 8N (PicoQuant). Absorption spectra were recorded on a Cary 60.

In the case of 2D Ag_12_-CAM, we observed a slight dependence of the emission spectra on the position on the crystal. In order to extract the main contribution to the observed emission, we performed a non-negative matrix factorization (using nnmf in Matlab) of the set of (a total of 40) experimental emission spectra. Preliminary single value decomposition (svd in Matlab) allowed us to assign 3 component spectra (with the eigenvalue of the most prominent being 10 times larger than the next one). The emission spectrum shown in the main manuscript represents the most prominent contribution of the three.

*Only data points recorded beyond the instrument response function (IRF) of the setup (approximately 2 ns) were included in the fitting. The time axis was rebinned from linear to logarithmic spacing with propagation of the associated errors. Nonlinear least-squares fitting was performed in Matlab using the sum of squared weighted residuals as the optimization criterion, (please check eq 10.31 of Molecular Fluorescence: Principles and Applications; Valeur, B.; Berberan-Santos, M. N., Eds.; Wiley-VCH: Weinheim, 2012).*

*The decay of 2D Ag_11_-CAM was fitted using a triple-exponential model:*

$I\left( t \right)=\sum_{i=1}^{3} A_{i}e^{-\frac{t}{\tau_{i}}}$	(Eq. S1)

*yielding τ₁ = 620 ns (A₁ = 21%), τ₂ = 6300 ns (A₂ = 33%), and τ₃ = 15800 ns (A₃ = 45%), with χ_r_²= 1.19 (Figure S16). Approximately 80% of the total luminescence intensity (A_i_t_i_) is associated with the longest component (τ₃ = 15.8 μs).*

*Any exponential model does not well describe the decay of 2D Ag_12_-CAM. It is instead fitted by a power-law function of the form:*

$I\left( t \right)=At^{-b}$	(Eq. S2)

*with b = 0.84 and χ_r_² = 10 (Figure S15). We note that the relatively elevated χ_r_² value is partly attributable to an afterpulsing artifact of the SPAD detector in the 100–1000 ns range, which distorts the data in this region and limits the quality of the fit.*

**Device fabrication.** To investigate the charge transport characteristics of the 2D Ag_12_-CAM and 2D Ag_11_-CAM materials, we fabricated lateral memristor devices, which stand as ITO/2D Ag_12_-CAM/ITO and ITO/2D Ag_11_-CAM/ITO, respectively. Both the devices were prepared by spin-coating slurries obtained after sonicating the as-synthesized crystals for one hour in isopropanol onto pre-patterned indium tin oxide (ITO) electrodes deposited on glass substrates. The coated films were subsequently dried at room temperature for several hours before being employed for electrical measurements.

**Transport measurement.** Transport measurements were conducted in a two-probe configuration using a Janis micromanipulated probe station (model ST-500) equipped with tungsten tips. The device was mounted inside a closed chamber, *which was evacuated to a base pressure of approximately 10⁻³ mbar*, and interfaced through LabVIEW to a Keithley 2634B source-measure unit (SMU). *The endurance and retention measurements were performed using a read pulse of 1 V with a pulse width of 200 ms*. Temperature-dependent conductivity measurements were performed using a 4 K cryostat fitted with an RDK-101D cold head operating on a two-stage Gifford–McMahon (GM) cycle cryo-refrigerator, controlled by a Lake Shore 336 temperature controller and interfaced through LabVIEW to a Keithley 2634B SMU. Single-crystal powders were pressed into pellets of size 5 × 5 mm² for further measurements.

**Computational details.**

**Study of the Ag*_x_*-Azopy-Ag*_x_* (*x* = 11, 12) model dimers.** Dimeric Ag*_x_*-azo-Ag*_x_* (*x* = 11, 12) fragments were extracted from the UNG2 and UNG33 crystal structures and their dangling bonds saturated with hydrogen atoms. The obtained dimers are closed-shell systems. Their structures have been optimized within DFT^[4]^ and the vibrational analyses performed on the optimized structures showed that the located extrema correspond to true minima (no imaginary frequencies). The calculations were performed with the Amsterdam Density Functional (ADF, version: 2026.103) program^[5]^ from the Amsterdam Modeling Suite (AMS, version: 2026.103)^[6]^. They were run spin-restricted and scalar-relativistically within the zero order regular approximation (ZORA) for relativistic effects^[7]^. The dispersion-corrected PBE-D3BJ^[8]^ functional was employed combined with a set of relativistic Slater-type orbital (STO) basis sets from the ADF basis set database^[9]^. In this set, the Ag atoms are described by a triple-ζ polarized (TZP) quality basis set, and the other atoms by a double-ζ polarized (DZP) quality basis set. Furthermore, the atomic core levels are kept frozen up to the 3d level for the Ag atoms, and up to the 1s level for the S, F, O, N and C atoms.

The visualization of the dimers’ structures and their frontier molecular orbitals (MOs) were made with the Jmol software^[10]^ and the associated MO level diagrams were drawn using the inkscape software.^[11]^.

**Table S1**. Crystal data and structure refinement for 2D Ag_12_-CAM.

Empirical formula	C_43_H_44_Ag_6_F_9_N_7_O_9_S_3_

Formula weight	1717.25

Temperature	119.99(10) K

Wavelength	1.54184 Å

Crystal system	Monoclinic

Space group	P 1 21/c 1

Unit cell dimensions	a = 15.14745(7) Å	α = 90°

b = 26.46937(10) Å	β = 108.7254(5)°

c = 14.96444(6) Å	γ = 90°

Volume	5682.31(4) Å^3^

Z	4

Density (calculated)	2.007 Mg/m^3^

Absorption coefficient	18.051 mm^-1^

F(000)	3336

Crystal size	0.291 x 0.233 x 0.148 mm^3^

Theta range for data collection	3.080 to 73.925°.

Index ranges	-16<=h<=18, -32<=k<=32, -18<=l<=18

Reflections collected	171229

Independent reflections	11364 [R(int) = 0.0634]

Completeness to theta = 67.684°	100.0 %

Absorption correction	Gaussian

Max. and min. transmission	0.480 and 0.041

Refinement method	Full-matrix least-squares on F^2^

Data / restraints / parameters	11364 / 491 / 883

Goodness-of-fit on F^2^	1.061

Final R indices [I>2sigma(I)]	R1 = 0.0417, wR2 = 0.1131

R indices (all data)	R1 = 0.0426, wR2 = 0.1139

Extinction coefficient	n/a

Largest diff. peak and hole	1.422 and -2.227 e.Å^-3^

**View of the asymmetric unit of 2D Ag_12_-CAM (displacement parameters at 50 percent probability level):**

**COMMENTS**

The ligand is disordered and was refined using two components. Geometrical restraints were added. Some trifluoromethyl groups are also disordered and were refined using 2 groups of three fluorine atoms, with geometrical restraints.

**Table S2**. Crystal data and structure refinement for 2D Ag_11_-CAM.

Empirical formula	C_95_H_73_Ag_11_F_51_N_13_O_15_S_6_

Formula weight	3984.59

Temperature	120.01(10) K

Wavelength	1.54184 Å

Crystal system	Monoclinic

Space group	P 1 21 1

Unit cell dimensions	a = 15.92427(11) Å	α = 90°

b = 26.46176(12) Å	β = 113.5362(8)°

c = 16.88013(12) Å	γ = 90°

Volume	6521.27(8) Å^3^

Z	2

Density (calculated)	2.029 Mg/m^3^

Absorption coefficient	15.053 mm^-1^

F(000)	3852

Crystal size	0.408 x 0.122 x 0.033 mm^3^

Theta range for data collection	2.855 to 76.363°.

Index ranges	-17<=h<=19, -32<=k<=33, -21<=l<=21

Reflections collected	190745

Independent reflections	26519 [R(int) = 0.0589]

Completeness to theta = 67.684°	100.0 %

Absorption correction	Gaussian

Max. and min. transmission	1.000 and 0.047

Refinement method	Full-matrix least-squares on F^2^

Data / restraints / parameters	26519 / 987 / 2015

Goodness-of-fit on F^2^	1.062

Final R indices [I>2sigma(I)]	R1 = 0.0614, wR2 = 0.1725

R indices (all data)	R1 = 0.0682, wR2 = 0.1800

Absolute structure parameter	0.349(11)

Extinction coefficient	n/a

Largest diff. peak and hole	3.862 and -1.732 e.Å^-3^

**View of the asymmetric unit of 2D Ag_11_-CAM (displacement parameters at 50 percent probability level):**

**COMMENTS:**

The model is imperfect:

Some displacement ellipsoids for the silver atoms are large. Some positions were split (Ag_9_, Ag_8_ and Ag_10_). A lot of CF_3_ groups in ligands and trifluoroacetate molecules are disordered. Nine of them were modelled using two components. Some restraints were used on some solvent molecules. There is residual density in the vicinity of Ag_8_. The DMA molecule close to this position may be slightly disordered. There is a residual peak in the open face of the cluster. This residual density appeared on all the datasets collected on different batches. Therefore, it is unlikely that it is just an artefact due to the crystal quality. The environment points to an extra Ag atom, partly occupied. The DMAc molecule that appears in the vicinity could then be partly replaced by a trifluoroacetate molecule, that would compensate for the extra charge. Such a model was attempted, but lots of restraints had to be used and the density being small, even if we could force this model, no evidence was made that it was true. (The electronic density around the DMAc molecule is pretty flat.) Other species than Ag are unlikely in view of the synthesis conditions.

The space group is a Sohncke group, but the Flack parameter is around 0.35. That could be an inversion twin. (The sources of possible chirality have not been explored.)

***Figure S1****. (a) OMIT map showing the residual density on the potential low-occupancy extra Ag atom. (b) OMIT map showing the electronic density on the DMAc molecule close to the possible extra Ag position.*

*The structure of 2D Ag_11_-CAM presents a residual density peak at a position geometrically consistent with the presence of an additional Ag atom with low occupancy (Figure S1a). Incorporation of this atom would require local charge compensation. As a DMAc molecule is located in close proximity to the residual density peak, one possible model is that this DMAc molecule is replaced by a CF₃COO⁻ ion in the presence of an extra Ag atom.*

*This alternative model was therefore investigated. When the isotropic displacement parameter of the additional Ag atom was fixed at 0.05 Å², refinement converged to an occupancy below 10%. Allowing the displacement parameter to refine freely resulted in an occupancy of approximately 14%. The model [[Ag_11_(C_8_F_6_H_3_S)_6_(CF_3_COO)_5_(C_3_H_7_NO)_3_(C_4_H_9_NO)_1_(C_10_H_8_N_4_)_2_].(C_4_H_9_NO)_1_]_0.9_[Ag_12_(C_8_F_6_H_3_S)_6_(CF_3_COO)_6_(C_3_H_7_NO)_3_(C_4_H_9_NO)_1_(C_10_H_8_N_4_)_2_]_0.1_ is provided as supplementary material. However, extra density peaks in Fourier maps close to the cluster core are common in Ag cluster structures because of the strong absorption of silver atoms. The OMIT map calculated for the DMAc molecule close to the possible Ag position was of excellent quality (Figure S1b), offering little crystallographic support for the disorder model involving replacement of DMAc by a CF₃COO⁻ anion, while the occupancy of the additional Ag site refined to a relatively low value. For these reasons, the simpler model containing only the Ag_11_ cluster was retained and is presented in the main text.*

**Figure S2.** (a) Four DMF molecules are coordinated to the Ag_12_ core of 2D Ag_12_-CAM, and (b) three DMF molecules and one DMAc molecule are coordinated to the Ag_11_ core of 2D Ag_11_-CAM.

**Figure S3**. The auxiliary ligand CF_3_COO^‒^ connected with (a) Ag_12_ core of 2D Ag_12_-CAM, and (b) Ag_11_ core of 2D Ag_11_-CAM.

**Figure S4**. The Azopy linkers connecting with (a) Ag_12_ core of 2D Ag_12_-CAM, and (b) Ag_11_ core of 2D Ag_11_-CAM.

*Figure S5. Interlayer stacking mode of 2D Ag_12_-CAM. (a) View showing the stacking of adjacent layers in an ABAB sequence. (b) View along [001] showing the lateral displacement of layer B (green) relative to layer A (blue).*

**Figure S6.** Adjacent layers of the 2D Ag_12_-CAM are interconnected mostly through C–F···H–C (~2.60 Å) and C–O···H–C (~2.68 Å) interactions, as analyzed using Mercury software.

*Figure S7. Interlayer stacking mode of 2D Ag_11_-CAM. (a) View showing the stacking of adjacent layers in an ABAB sequence. (b) View along [-1 0 -1] showing the lateral displacement of layer B (green) relative to layer A (blue).*

**Figure S8**. Adjacent layers of the 2D Ag_11_-CAM are interconnected mostly through C–F···H–C (~2.41 Å), C–F···F–C (~2.82 Å), and C–H···H–C (~2.17 Å) interactions, as analyzed using Mercury software.

**Figure S9**. Structural view of π–π stacking interactions within each sheet of 2D Ag_11_-CAM between the aromatic ring of Azopy linkers and BTFMBT ligands, showing an average centroid–centroid distance of 3.67 Å, which contributes to framework stabilization (analyzed by using Olex2 software).

**Figure S10**. Powder X-ray diffraction pattern of (a) 2D Ag_12_-CAM, *(b) 2D Ag_11_-CAM*.

***Figure S11****. Molecular orbital energy diagram of the cluster–linker–cluster model representing 2D Ag_12_-CAM, showing the spatial distribution of selected frontier orbitals (HOMO to HOMO-4 and LUMO to LUMO+2).*

***Figure S12****. Molecular orbital energy diagram of the cluster–linker–cluster model representing 2D Ag_11_-CAM, showing the spatial distribution of selected frontier orbitals (HOMO to HOMO-4 and LUMO to LUMO+2).*

**Figure S13**. TGA analysis of 2D Ag_12_-CAM and 2D Ag_11_-CAM.

**Figure S14**. Contact angle measurement for (a) 2D Ag_12_-CAM and (b) 2D Ag_11_-CAM.

***Figure S15****: Luminescence decay of 2D Ag_12_-CAM and the corresponding power-law fit. The upper panel shows the weighted residuals.*

***Figure S16****: Luminescence decay of 2D Ag_11_-CAM and the corresponding triple-exponential fit. The upper panel shows the weighted residuals.*

**Figure S17**. Variation of resistivity as a function of temperature for (a) 2D Ag_12_-CAM and (b) 2D Ag_11_-CAM.

**Figure S18**. Patterned ITO/glass substrate showing two parallel ITO electrodes separated by an etched glass gap (~200 μm), defining the lateral device geometry.

***Figure S19****. Comparison of the simulated PXRD patterns of (a) 2D Ag_12_-CAM and (b) 2D Ag_11_-CAM with those of the corresponding samples recovered after 1 h sonication in isopropanol. Intensities were normalized to the most intense diffraction peak for ease of comparison.*

***Figure S20****. High-resolution Ag 3d XPS spectra of (a) 2D Ag_12_-CAM and (b) 2D Ag_11_-CAM recorded before and after device fabrication. High-resolution O 1s XPS spectra of (a) 2D Ag_12_-CAM and (b) 2D Ag_11_-CAM recorded before and after device fabrication.*

**Figure S21**. Small-signal I–V characteristics of device D-1: (a) Forward-sweep small signal I–V curves exhibiting pronounced current asymmetry favouring positive bias. (b) Reverse-sweep small-signal I–V curves displaying stronger conduction under negative bias. The bias-dependent asymmetry captured in both figures unambiguously demonstrates diode behaviour.

**Figure S22**. Double-logarithmic plots with corresponding linear fits for device D-1: (a) Path 1 during the forward voltage sweep, (b) Path 3 in the forward sweep, (d) Path 4 in the reverse sweep, (e) Path 6 in the reverse sweep. (c) Negative differential resistance (NDR) behavior under positive bias, and (f) NDR behavior under negative bias.

**Figure S23**. Small-signal I–V characteristics of device D-2: (a) Forward-sweep small signal I–V curves exhibiting pronounced current asymmetry favouring positive bias. (b) Reverse-sweep small-signal I–V curves displaying stronger conduction under negative bias. The bias-dependent asymmetry captured in both figures unambiguously demonstrates diode behaviour.

**Figure S24**. Double-logarithmic plots with corresponding linear fits for device D-2 (a) Path 1 during the forward voltage sweep, (b) Path 3 in the forward sweep, (d) Path 4 in the reverse sweep, and (e) Path 6 in the reverse sweep. (c) NDR behavior under positive bias, and (f) NDR behavior under negative bias.

**Figure S25**. I–V plot illustrating peak-to-valley ratio (PVR) and switching efficiency (SE) of the devices (a) D-1 and (b) D-2.

***Figure S26****. Current–voltage (I–V) characteristics of control devices fabricated from the individual framework components: (a) Ag–BT complex, (b) Ag–BTFMBT complex, and (c) Azopy linker, measured under the same device geometry and fabrication conditions as those used for devices D-1 and D-2. Measurements were reproduced using multiple independently fabricated control devices.*

*Figure S27. Device-to-device reproducibility of the 2D Ag_12_-CAM (D-1) memristive devices. Representative current–voltage (I–V) characteristics of 20 independently fabricated devices measured under identical fabrication and testing conditions.*

*Figure S28. Device-to-device reproducibility of the 2D Ag_11_-CAM (D-2) memristive devices. Representative current–voltage (I–V) characteristics of 20 independently fabricated devices measured under identical fabrication and testing conditions.*

***Figure S29****. Retention characteristics of (a) D-1 and (b) D-2 measured at room temperature (RT) and 333 K as a function of time using a 1 V read pulse with a pulse width of 200 ms.*

*Figure S30. Long-term ambient stability of the memristive devices after storage under ambient laboratory conditions (22–27 °C) for more than five months. (a,b) Resistive-switching characteristics and (c,d) corresponding semi-logarithmic current–voltage (I–V) curves of devices D-1 and D-2 measured after prolonged ambient exposure.*

**Artificial Neural Network training and MNIST recognition:**

A single-layer artificial neural network (ANN) was implemented in PyTorch^[12]^ to classify handwritten digits from the Modified National Institute of Standards and Technology (MNIST) dataset, employing conductance data derived from the P/D characteristic under a presynaptic voltage pulse of 1 V. The network architecture comprised 784 input neurons, corresponding to each pixel in the 28 × 28 greyscale MNIST image, and 10 output neurons representing digits 0–9 (Figure 6a).

Each input image was flattened into a vector of 784 input neurons, which were directly connected to the output layer through synaptic weights. The weighted inputs were summed and passed through a SoftMax activation function to introduce nonlinearity. During training, the predicted outputs were compared against the ground truth labels, with the mean cross-entropy function employed as the loss metric. Weight updates were performed using the Adaptive Moment Estimation (Adam) optimizer with a learning rate of 0.1. This iterative process of minimizing error and adjusting weights via the Adam algorithm constitutes the backpropagation method.

Training was conducted on the full MNIST dataset of 60,000 images, divided into mini-batches of 32 to enable efficient GPU-based computation. Each complete pass through the dataset was defined as one epoch. Following training, the model was evaluated on 10,000 unseen test images, with accuracy computed as the ratio of correct predictions to total test samples.

Once the software-based model was trained, the learned weights were quantized using an adaptive quantization scheme with 50 conductance levels. These quantized weights were subsequently mapped to the synaptic device by modulating the number of applied voltage pulses.

**Conductance Model and Weight Mapping.** To model the conductance behaviour of synaptic devices, the amount of conductance change (δG) plays an important role. For long-term potentiation (LTP) and long-term depression (LTD), the *δG* equations are represented as follows:

(Eq. S3)

(Eq. S4)

Where *δG* is the amount of conductances change for a given conductance*. G_max_* and *G_min_* are the maximum and minimum conductance measured from the synaptic device respectively. *α* is a fitting parameter and *β* is a nonlinearity factor

The conductance behaviour of the synaptic device was modelled as:

$G\left( x \right)=p+qln(x+r)$	(Eq. S5)

where *G* is the conductance, *x* is the number of applied pulses to the device, p and *r* are fitting parameters, and *q* represents the nonlinearity factor. The model demonstrated excellent agreement with experimental conductance data.

For inference, the adaptive quantization method was employed to incorporate device nonlinearity, thereby reflecting the intrinsic characteristics of the synaptic element. Prior to mapping, the trained weights were normalized to the conductance range of the device. The number of pulses required for mapping was calculated as:

$x=round[\exp(\frac{|w|-p}{q})-r]$	(Eq. S6)

where *x* is the pulse count and *|w|* is the absolute value of the software-trained weight. Since conductance is inherently positive, differential mapping was adopted to represent both positive and negative synaptic weights. In this scheme, each weight was encoded using two devices, with the effective weight defined as *w = (G^+^ - G^-^),* where *G*^+^ and *G*^-^ denote the conductance values of the paired devices. Device-level test accuracy was evaluated using this differential mapping approach.^[13]^

**Accuracy and Resolution Dependence.** The ANN maintained accuracy across multiple epochs, with the device-based implementation achieving an average accuracy of ~84 %, compared to ~93% for the purely software-based model (Figure 6b). The high fidelity of the device-level ANN is attributed to both the robust performance of the synaptic device and the efficient weight-mapping strategy.

To further assess scalability, the model was tested with reduced input image resolutions (Figure S32). Lower resolution inputs are particularly relevant for resource-constrained applications such as Internet of Things (IoT) sensors, traffic monitoring systems, and compact smart devices, where memory and storage are limited. As expected, accuracy decreased with reduced resolution; however, even at 8 × 8 pixels, the device-based ANN achieved accuracy exceeding 70%. This result underscores the suitability of the synaptic device for neuromorphic hardware implementations in real-world scenarios.

**Figure S31**. Current behaviour of the 2D Ag_11_-CAM with respect to the number of identical pulses and its fit curve using a model Eq. S5.

**Figure S32**. Accuracy evaluation of the quantization methods with the variation of the input MNIST image resolutions.

***Hardware-Aware ANN Evaluation.*** *To further evaluate the robustness of the proposed hardware inference, additional experimentally measured device D-2 characteristics using (4V, 50ms) were incorporated into the hardware-aware model (Figure S33a).* *The software ANN achieves a classification accuracy of 92.36%, while the experimentally calibrated hardware model achieves 82.53% without considering cycle-to-cycle (C2C) variability and asymmetric potentiation/depression characteristics. Incorporation of these experimentally measured device non-idealities into the hardware model reduces the classification accuracy to 77% (Figure S33b-S33c), providing a more realistic representation of practical device operation. The reduction in accuracy primarily arises from conductance quantization, cycle-to-cycle variability, and nonlinear synaptic weight updates, which increase the mismatch between the ideal software-trained weights and their hardware representation. The corresponding confusion matrix of the experimentally informed hardware model is shown in Figure S33d.*

***Sparse-coding image reconstruction.*** *To further demonstrate the neuromorphic capability of the device D-2, an image reconstruction task was performed using sparse-coding simulations. In this approach, a software dictionary was trained using 500 MNIST images with a 4 × 4 pixel patch size and a sparsity parameter of λ = 0.1 over 40 epochs. The trained dictionary was then mapped onto the device by incorporating the experimentally measured device parameters. The software implementation achieves a mean squared error (MSE) of 0.00255, whereas the device-aware implementation achieves an MSE of 0.00450. The slightly higher MSE of the device-aware implementation arises from weight quantization errors. Despite this increase, the reconstruction error remains low, indicating that the reconstructed images closely resemble the software-generated and original images, as illustrated in Figure S34. These results further highlight the potential of the proposed memristive device for neuromorphic applications.*

***Figure S33****. Neuromorphic performance of the 2D Ag_11_-CAM-based device incorporating experimentally measured device non-idealities. (a) Experimentally measured potentiation/depression characteristics used for hardware-aware ANN simulations. (b) Cycle-to-cycle conductance variability extracted from repeated synaptic updates. (c) Comparison of software accuracy and hardware-aware accuracy after incorporating experimentally measured device non-idealities. (d) Confusion matrix for MNIST classification obtained using the experimentally informed hardware mode.*

***Figure S34.*** *Comparison of original MNIST digit images with their corresponding software-based and memristor-mapped sparse coding reconstructions. The dictionary was trained offline using sparse coding on non-overlapping 4 × 4 image patches extracted from the MNIST training dataset and subsequently mapped onto experimentally measured memristor conductance states using the experimentally derived LTP/LTD characteristics. Six representative examples of the digit '3' are shown. The mean squared error (MSE) between each reconstructed image and its corresponding original image is indicated above the reconstructed images. The memristor-mapped implementation exhibits a slightly higher MSE than the software implementation owing to conductance quantization during hardware mapping.*

***Energy Consumption.*** *To quantitatively evaluate the energy efficiency of the proposed artificial synapse, we have included the operating current, power consumption, and area-normalized energy consumption per synaptic update in the revised manuscript. Under the employed programming conditions (4 V, 50 ms pulse width), the device operates at an average current of approximately 418 pA, corresponding to a power consumption of approximately 1.67 nW and an estimated energy consumption of 6.63 pJ mm⁻² per synaptic update.*

**Table S3**. Hyperparameters and their description used for ANN simulations

| Hyperparameter | Names/Values |
| --- | --- |
| Learning rate | 0.1 |
| Batch size | 32 |
| Bias | No |
| Training epochs | 100 |
| Activation function | SoftMax |
| Loss function | Cross-entropy loss |
| Optimiser | Adaptive Moment Estimation (Adam) |

**Table S4**. Benchmark comparison of the neuromorphic performance of the as-synthesised 2D Ag_11_-CAM memristive devices with representative Ag-based cluster materials, metal–organic framework (MOF)-based, oxide-based, and other low-dimensional memristive systems reported in the literature.

| **Materials** | **Non–linearity**  **(LTP/LTD)** | **Asymmetry** | **Energy per synaptic update/ Power Consumption** | **On/Off Ratio** | **Endurance**  **(Cycle)** | **Retention (s)** | **Accuracy** | **References** |
| --- | --- | --- | --- | --- | --- | --- | --- | --- |
| PEA_2_MA*_n_*_−1_Pb*_n_*I_3_*_n_*_+1_  memristive device | 0.07/0.1 | - | - | - |  | - | 96.5% | ^[14]^ |
| Au/MoTe_2_/Ti/Au | 0.006/0.015 | - | - | 10-10^4^ | - | 10^4^ s | 96.05% | ^[15]^ |
| Au/PMMA/BDAMA*_n_*_−1_Pb*_n_*I_3_*_n_*_+1_/ITO | 0.002/  -0.0015 | 0.0005 | 2.81pJ | - | - | 10^4^ s | 98.75% | ^[16]^ |
| MoS_2_/Nb_2_O_5_/Al_2_O_3_ | 0.81/2.18 | 0.39 (Asymmetry ratio) | 6pJ | 30 | 200 | 10^3^ s | 94.21% | ^[17]^ |
| Au/h-BN/Au | - | - | - | 10^7^ | >300 | >2 × 10^4^ | - | ^[18]^ |
| Pt/Ti_3_C_2_/Pt | - | - | 1mJ | 10^3^ | 2000 | 10^4^ s | 79.8-80.6% | ^[19]^ |
| 1 D Ag_12_ -CAM | - | - | - | 70 | 1000 | 1000 s | - | ^[20]^ |
| 0 D Ag_12_ -NC | - | - | - | 13 | 1000 | 1000 s | - | ^[20]^ |
| Ag/MoTe_2_/  ITO | - | - | 74.2 pJ |  | 10^4^ | 10^4^ | - | ^[21]^ |
| Ag/Rb-CD-MOF/Ag | - | - | - | 150 | - | 600 s | - | ^[22]^ |
| Pt/RSMOF-1/W | - | - | 0.4nW or 0.2pW | 30 | - | 6000 s | - | ^[23]^ |
| Au/HKUST-1/Au/PET | - | - | - | 10 | - | 10,000 s | - | ^[24]^ |
| Cu/HKUST-1/Cu | - | - | - | 5 | - | 24 h | - | ^[25]^ |
| Cu/ferrocene-(KUST-1)/Au | - | - | - | 60 | - | - | - | ^[26]^ |
| SiO_2_/[Cu_2_I_2_(TAA)]_n_/SiO_2_ | - | - | - | 0.1 | - | - | - | ^[27]^ |
| Ag/MIL-53/GaInSn@ PDMS | - | - | - | 200 | - | 100,000 s | - | ^[28]^ |
| Pd/ZnO@ZIF-8/Pt | 0.58%/  0.30%,  0.51%/  0.19% | 1.008/1.014 (Asymmetry ratio) | - | 44.54 | - | - | - | ^[29]^ |
| Pt/MIL-53-H/ITO | - | - | - | 25 | - | 1000s | - | ^[30]^ |
| **2D Ag_11_-CAM** | **3.78/-10.41** | **0.168** | **6.63 pJmm^-2^** | **642** | **4000** | - | **77%** | **This work** |

**References**.

[1]	O. V. Dolomanov, L. J. Bourhis, R. J. Gildea, J. A. Howard, H. Puschmann, *Applied Crystallography* **2009**, *42*, 339-341.

[2]	G. M. Sheldrick, *Foundations of Crystallography* **2015**, *71*, 3-8.

[3]	L. Zhao, J. L. P. Lustres, V. Farztdinov, N. P. Ernsting, *Physical Chemistry Chemical Physics* **2005**, *7*, 1716-1725.

[4]	aP. Hohenberg, W. Kohn, *Physical review* **1964**, *136*, B864; bW. Kohn, L. J. Sham, *Physical review* **1965**, *140*, A1133.

[5]	(a) E. J. Baerends, N. F. Aguirre, N. D. Austin, J. Autschbach, F. M. Bickelhaupt, R. Bulo, C. Cappelli, A. C. van Duin, F. Egidi, C. Fonseca Guerra, *The Journal of chemical physics* **2025**, *162*; (b) SCM, ADF 2026.1, Theoretical Chemistry, Vrije Universiteit, Amsterdam, The Netherlands, <https://www.scm.com>., E.J. Baerends, T. Ziegler, A.J. Atkins, J. Autschbach, O. Baseggio, D. Bashford, A. Bérces, F.M. Bickelhaupt, C. Bo, P.M. Boerrigter, C. Cappelli, L. Cavallo, C. Daul, D.P. Chong, D.V. Chulhai, L. Deng, R.M. Dickson, J.M. Dieterich, F. Egidi, D.E. Ellis, M. van Faassen, L. Fan, T.H. Fischer, A. Förster, C. Fonseca Guerra, M. Franchini, A. Ghysels, A. Giammona, S.J.A. van Gisbergen, A. Goez, A.W. Götz, J.A. Groeneveld, O.V. Gritsenko, M. Grüning, S. Gusarov, F.E. Harris, P. van den Hoek, Z. Hu, C.R. Jacob, H. Jacobsen, L. Jensen, L. Joubert, J.W. Kaminski, G. van Kessel, C. König, F. Kootstra, A. Kovalenko, M.V. Krykunov, P. Lafiosca, E. van Lenthe, D.A. McCormack, M. Medves, A. Michalak, M. Mitoraj, S.M. Morton, J. Neugebauer, V.P. Nicu, L. Noodleman, V.P. Osinga, S. Patchkovskii, M. Pavanello, C.A. Peeples, P.H.T. Philipsen, D. Post, C.C. Pye, H. Ramanantoanina, P. Ramos, W. Ravenek, M. Reimann, J.I. Rodríguez, P. Ros, R. Rüger, P.R.T. Schipper, D. Schlüns, H. van Schoot, G. Schreckenbach, J.S. Seldenthuis, M. Seth, J.G. Snijders, M. Solà, M. Stener, M. Swart, D. Swerhone, V. Tognetti, G. te Velde, P. Vernooijs, L. Versluis, L. Visscher, O. Visser, F. Wang, T.A. Wesolowski, E.M. van Wezenbeek, G. Wiesenekker, S.K. Wolff, T.K. Woo, A.L. Yakovlev.

[6]	M. F. R. Rüger, T. Trnka, A. Yakovlev, E. van Lenthe, P. Philipsen, T. van Vuren, B. Klumpers, T. Soini. AMS 2026.1, SCM, Theoretical Chemistry, Vrije Universiteit, Amsterdam, The Netherlands, <https://www.scm.com>.

[7]	aE. v. Lenthe, E.-J. Baerends, J. G. Snijders, *The Journal of chemical physics* **1993**, *99*, 4597-4610; bE. van Lenthe, E.-J. Baerends, J. G. Snijders, *The Journal of chemical physics* **1994**, *101*, 9783-9792; cE. Van Lenthe, A. Ehlers, E.-J. Baerends, *The Journal of chemical physics* **1999**, *110*, 8943-8953.

[8]	aJ. P. Perdew, K. Burke, M. Ernzerhof, *Physical review letters* **1996**, *77*, 3865; bS. Grimme, J. Antony, S. Ehrlich, H. Krieg, *The Journal of chemical physics* **2010**, *132*; cS. Grimme, S. Ehrlich, L. Goerigk, *Journal of computational chemistry* **2011**, *32*, 1456-1465.

[9]	E. Van Lenthe, E. J. Baerends, *Journal of computational chemistry* **2003**, *24*, 1142-1156.

[10]	(a) p <http://www.jmol.org/>; (b) R. M. Hanson, *Applied Crystallography* **2010**, *43*, 1250-1260; cR. M. Hanson, J. Prilusky, Z. Renjian, T. Nakane, J. L. Sussman, *Israel Journal of Chemistry* **2013**, *53*, 207-216.

[11]	p. <https://inkscape.org/>.

[12]	K. Wang, Z. Liu, Y. Lin, J. Lin, S. Han, in *Proceedings of the IEEE/CVF conference on computer vision and pattern recognition*, **2019**, pp. 8612-8620.

[13]	D. Kwon, S. Lim, J.-H. Bae, S.-T. Lee, H. Kim, C.-H. Kim, B.-G. Park, J.-H. Lee, *IEEE Transactions on Electron Devices* **2018**, *66*, 395-401.

[14]	S. J. Kim, T. H. Lee, J.-M. Yang, J. W. Yang, Y. J. Lee, M.-J. Choi, S. A. Lee, J. M. Suh, K. J. Kwak, J. H. Baek, *Materials Today* **2022**, *52*, 19-30.

[15]	J. Yang, A. Yoon, D. Lee, S. Song, I. J. Jung, D. H. Lim, H. Jeong, Z. Lee, M. Lanza, S. Y. Kwon, *Advanced Functional Materials* **2024**, *34*, 2309455.

[16]	S. J. Kim, I. H. Im, J. H. Baek, S. Choi, S. H. Park, D. E. Lee, J. Y. Kim, S. Y. Kim, N.-G. Park, D. Lee, *Nature Nanotechnology* **2025**, *20*, 83-92.

[17]	J. H. Nam, S. Oh, H. Y. Jang, O. Kwon, H. Park, W. Park, J. D. Kwon, Y. Kim, B. Cho, *Advanced Functional Materials* **2021**, *31*, 2104174.

[18]	J. Y. Mao, S. Wu, G. Ding, Z. P. Wang, F. S. Qian, J. Q. Yang, Y. Zhou, S. T. Han, *Small* **2022**, *18*, 2106253.

[19]	X. Zhang, H. Chen, S. Cheng, F. Guo, W. Jie, J. Hao, *ACS applied materials & interfaces* **2022**, *14*, 44614-44621.

[20]	N. Alam, T. Rahaman, A. K. Das, A. K. Pal, A. Datta, S. J. Ray, P. K. Mondal, M. Polentarutti, S. Mandal, *Small* **2025**, *21*, 2409118.

[21]	T. Yu, Z. Zhao, H. Jiang, Z. Weng, Y. Fang, Y. Yu, W. Lei, S. Shafe, M. Mohtar, *Materials Today Chemistry* **2023**, *27*, 101268.

[22]	S. M. Yoon, S. C. Warren, B. A. Grzybowski, *Angewandte Chemie International Edition* **2014**, *53*, 4437-4441.

[23]	L. Pan, G. Liu, H. Li, S. Meng, L. Han, J. Shang, B. Chen, A. E. Platero-Prats, W. Lu, X. Zou, *Journal of the American Chemical Society* **2014**, *136*, 17477-17483.

[24]	L. Pan, Z. Ji, X. Yi, X. Zhu, X. Chen, J. Shang, G. Liu, R. W. Li, *Advanced Functional Materials* **2015**, *25*, 2677-2685.

[25]	S. D. Worrall, M. A. Bissett, W. Hirunpinyopas, M. P. Attfield, R. A. Dryfe, *Journal of Materials Chemistry C* **2016**, *4*, 8687-8695.

[26]	Z. Wang, D. Nminibapiel, P. Shrestha, J. Liu, W. Guo, P. G. Weidler, H. Baumgart, C. Wöll, E. Redel, *ChemNanoMat* **2016**, *2*, 67-73.

[27]	M. Moreno-Moreno, J. Troyano, P. Ares, O. Castillo, C. A. Nijhuis, L. Yuan, P. Amo-Ochoa, S. Delgado, J. Gómez-Herrero, F. Zamora, *ACS nano* **2018**, *12*, 10171-10177.

[28]	X. Yi, Z. Yu, X. Niu, J. Shang, G. Mao, T. Yin, H. Yang, W. Xue, P. Dhanapal, S. Qu, *Advanced Electronic Materials* **2019**, *5*, 1800655.

[29]	C.-M. Yeom, D. Kumar, S. B. Eadi, H.-S. Lee, P. K. Thallapally, H.-M. Kwon, R. A. Fischer, H.-D. Lee, K. Jayaramulu, *Cell Reports Physical Science* **2024**, *5*.

[30]	K. Chen, Z. Wang, Z. Yang, Y. Liu, X. Huang, J. Yue, *physica status solidi (a)* **2025**, *222*, 2400801.
